# Disparities in statin use in patients with ASCVD with vs without rheumatologic diseases in a large integrated healthcare system: Houston methodist CVD learning health system registry

**DOI:** 10.1016/j.ajpc.2025.100959

**Published:** 2025-03-28

**Authors:** Eleonora Avenatti, Helene DiGregorio, Elia El Hajj, Rakesh Gullapelli, Kenneth Williams, Izza Shahid, Budhaditya Bose, Kobina Hagan, Juan C Nicolas, Shubham Lahan, Nwabunie Nwana, Sara Ayaz Butt, Kanika Monga, Lily Anne Romero Karam, Myriam Guevara, Zulqarnain Javed, Brittany Weber, Sadeer Al-Kindi, Khurram Nasir

**Affiliations:** a Department of Cardiology, Houston Methodist DeBakey Heart & Vascular Center, Division of Cardiovascular Prevention, Houston Methodist Academic Institute, 6550 Fannin St Suite 1101, Houston, TX 77030, USA; b Department of Internal Medicine, Houston Methodist Hospital, Houston, TX, USA; c Center for Health Data Science and Analytics, Houston Methodist, Houston, TX, United States; d Department of Medicine, Division of Rheumatology, Houston Methodist Hospital, Houston, TX, USA; e Department of Cardiovascular Medicine, Brigham and Women's Hospital, Boston, MA, USA

**Keywords:** Cardiovascular disease, Rheumatological disorders, Cardiovascular risk, Gender disparities, Statins, Health equity, Cardio-rheumatology

## Abstract

**Objective:**

The comorbid presence of Rheumatologic Diseases (RDs) and Atherosclerotic Cardiovascular Disease (ASCVD) substantially accentuates cardiovascular risk. We aimed to compare rates of secondary prevention statin utilization in patients with established ASCVD both with and without underlying comorbid RDs– and to highlight any potential gender, racial, or ethnic disparities in statin use in a contemporary US cohort.

**Methods:**

We queried the electronic medical record (EHR)-linked Houston Methodist Learning Health System Outpatient Registry containing data for approximately 1.2 million patients to identify patients with diagnosed ASCVD and RDs using ICD-10 codes. Statin prescription rates and dosage were evaluated via ATC codes.

**Results:**

Among 113,021 patients with ASCVD, 7286 (6.4 %) had comorbid RDs. The majority (71.1 %) of patients with ASCVD were prescribed statins, with discernibly lower utilization in patients with comorbid RDs compared to the non-RD population (63.2 % vs. 71.7 %, *p* < 0.005). High-intensity statins were prescribed in 42,636 (37.7 %) of ASCVD patients, with similarly reduced utilization in RD vs non-RD patients (30.4 % vs. 38.2 %). These trends remained consistent across sociodemographic subgroups. Moreover, women were consistently less likely to receive high intensity statins in both RD and non-RD groups. Reduced statin utilization was not accounted for with non-statin lipid lowering therapies in RD vs non RD subgroups.

**Conclusion:**

In this real-world study, co-morbid RDs were associated with significant lower utilization of secondary prevention statin therapy in patients with ASCVD. A multidisciplinary team approach may help to better understand key drivers of statin uptake in this clinically vulnerable population.

**Significance and innovation**

• The comorbid presence of Rheumatologic Diseases (RDs) and Atherosclerotic Cardiovascular Disease (ASCVD) accentuates cardiovascular risk substantially.

• Statin use is an evidence-based secondary prevention strategy that can effectively reduce the risk of recurrent ASCVD events. Despite this, these medications are underutilized for patients with comorbid RD.

• Statin utilization patterns for secondary ASCVD prevention in a large contemporary cohort of patients with and without RDs highlights considerable disparities in care and potential treatment gaps that may be addressed by a holistic, multidisciplinary approach.

Alt-text: Unlabelled box

## 1 Background

Atherosclerotic cardiovascular disease (ASCVD) is the leading cause of morbidity and mortality worldwide and in the US [1]. Despite significant progresses in medical management, patients with established disease are at a high risk for recurrent events[2].

Patients with RDs are at a high risk for ASCVD due to a multitude of factors, including chronic inflammation, which is an established mediator in the pathophysiology of atherosclerosis [3,4]. Other factors, including the iatrogenic effects of medications, the impact of RDs on mobility and disability, and the higher prevalence of shared ASCVD risk factors all contribute to the increased risk of ASCVD in patients with RDs.

Statins are the cornerstone of lipid lowering treatment and are among the primary pharmacological tools available to reduce residual ASCVD risk. Statin use in ASCVD for secondary prevention is widely endorsed based on numerous clinical trials and has a class I recommendation in international guidelines [5,6]. Patients with RDs should undergo lipid lowering therapies based on current recommendations for the general population [7,8]^.^ In addition to the well-recognized lipid-lowering effects, statins also exert anti-inflammatory and immunomodulatory pleiotropic properties, which could provide added benefits for patients with ASCVD and concomitant RDs [9]. To date, no large studies have explored the use of statin therapies among patients with RDs.

Examining trends and patterns of statin utilization in a real-world, contemporary population of ASCVD patients, both with and without RDs, may highlight current gaps in care and inform evidence-based pharmacotherapeutic management for this high-risk population.

In this study, we analyzed the rates of secondary prevention statin utilization among patients with established ASCVD both with and without comorbid RDs in a large contemporary US cohort to identify areas where targeted interventions and multidisciplinary approaches are most necessary to ensure optimal care for this clinically vulnerable population.

## 2 Methods

### 2.1 Setting and study design

The Houston Methodist Cardiovascular Disease Learning Health System Registry (CVD-LHS), an Electronic Medical Record (EMR) linked database that collects de-identified data of all adult patients who were seen in one of the HMH outpatient facilities, was queried for this observational cross-sectional study. The registry includes patients’ demographics, diagnoses, test results (blood, imaging), prescriptions, and information on comorbidities and social determinants of health (SDOH).

The Houston Methodist Institutional Review Board reviewed and approved the study protocol.

#### 2.1.1 Study population

The CVD-LHS Registry was used to identify all patients age ≥18 years with at least one outpatient encounter at HMH from June 2016 until April 2022. Patients with a diagnosis of established ASCVD (coronary artery disease (CAD), peripheral artery disease (PAD), or non-hemorrhagic stroke (CVA)) were included in the study. Diagnoses were established based on previously validated definitions [10,11] and extracted from the patient's chart using the appropriate International Classification of Diseases-Tenth Revision-Clinical Modification codes (ICD-10) (see supplemental Tables 1–3 for a detailed list). Patients with RDs were identified similarly using ICD-10 codes as selected by clinical experts from HMH Rheumatology Division. The following conditions were included in the Rheumatologic Disease Cohort: Rheumatoid arthritis (RA), Juvenile RA, Ankylosing Spondyloarthropathies including Psoriatic Arthritis (SP-A), Systemic Lupus Erythematosus (SLE), Systemic Sclerosis (SS), Sjogren Syndrome (SjS), Mixed connective tissue disease, and Inflammatory Myopathies (see suppl Tables for the complete list of included ICD-10 codes).

#### 2.1.2 Outcomes of interest

The primary outcome of interest was the prescription rates of statins among ASCVD patients, with and without RDs. We reported both overall statin prescriptions and prescriptions categorized into low/moderate and high-intensity statins groups. Statin use and prescribed dosage were retrieved from the Registry based on medical reconciliation reviews completed at each clinical encounter. This information was gathered following the current Anatomic Therapeutic Chemical (ATC) classification. If a patient had multiple recorded encounters in the registry, data from the most recent encounter were used. Recorded Statin usage included prescriptions of atorvastatin, simvastatin, lovastatin, pravastatin, fluvastatin, cerivastatin, rosuvastatin, and pitavastatin. Statin intensity was defined in accordance with the ACC AHA 2013 guidelines [5].

#### 2.1.3 Covariates

Other variables of interest included gender (male vs female, self-reported), race and ethnicity (self-reported; non-Hispanic White (NHW), non-Hispanic Black (NHB), Asian and Hispanic), age (<40; 40–64 and ≥65 years), and socioeconomic cardiovascular risk profile. Socioeconomic disadvantage was captured through insurance status (insured vs uninsured) and quintiles of ADI (Area Deprivation Index - a validated tool describing socioeconomic disadvantage of a specific area based on income, education, employment and housing quality). For each patient, insurance information was retrieved from the EMR, and the appropriate ADI quintile was assigned based on their mailing address zip code. Cardiovascular comorbidities included established ASCVD RF including diabetes mellitus (DM), obesity, smoking, hypertension, chronic kidney disease, and heart failure (all captured through the EMR with the appropriate ICD-10 codes).

#### 2.1.4 Statistical analysis

Continuous variables were presented as means +/- Standard deviation (SD) or median with interquartile range (IQ) depending on normality. Categorical variables are presented as frequencies and percentages. Prevalence of statin prescriptions and statin intensity were assessed amongst patients with ASCVD with and without RDs, and in women vs men in the former group with Pearson χ2 test.

To evaluate the association between comorbid RDs and statin use in patients with ASCVD, as well as the impact of gender on statin uptake among patients with ASCVD and RDs, multivariable logistic regression analysis was used to control for possible confounders. In model 1 the analysis was adjusted for baseline demographics: age and race/ethnicity. In model 2 we further adjusted for socioeconomic and cardiovascular risk factors as described above and use of non-statin lipid-lowering medications (ezetimibe, bempedoic acid, PCSK9-inhibitors, fibrates, omega-3 fatty acids, cholesterol absorption inhibitors). Adjusted odds ratio (OR) with 95 % confidence intervals (CI) were calculated for both models, determining the odds of any statin use (subdivided into low-moderate intensity statin use or high-intensity statin use) among ASCVD patients with and without comorbid RDs, as well for women compared with men among patients with ASCVD and RDs.

All analyses were conducted using STATA (StataCorp Version 17). *p* value <0.05 was considered significant in all instances.

## 3 Results

**Fig. 1:**
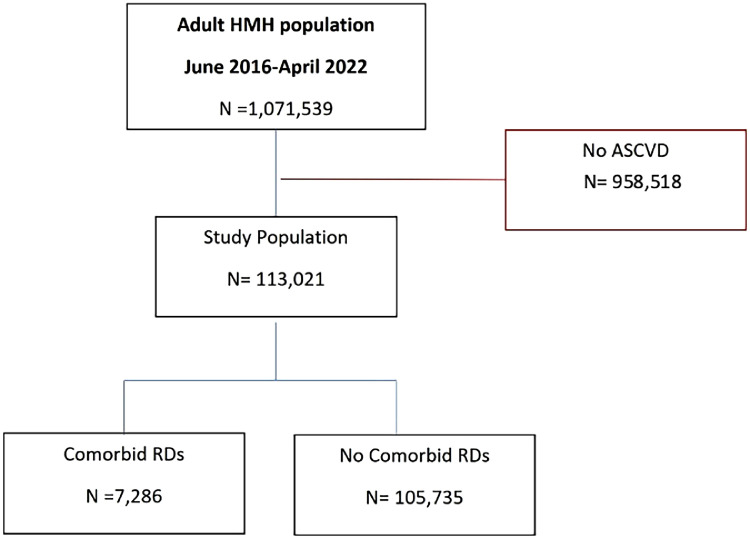
Flowchart detailing inclusion of study population based on eligibility criteria. HM, Houston Methodist; ASCVD, Atherosclerotic Cardiovascular Disease.

Among 1,071,539 adult patients seen in the outpatient setting at Houston Methodist Hospital between June 2016 and April 2022, a total of 113,021 had an established diagnosis of ASCVD (CAD, PAD, or CVA). Among these, 7286 patients (6.4 %) had a concurrent diagnosis of RDs (Fig. 1).

Compared to patients without RDs, patients with RDs were more likely to be female (70.9 % vs 43 %, *p* < 0.01) while age and race/ethnicity appeared to be overall similar within the two groups, with the majority of patients being > 65 years of age and identifying as Non-Hispanic White, although Non-Hispanic Black American were proportionally more represented in the RD population (Table 1). The RDs group also had slightly higher rates of higher ADI quintiles, indicating higher socioeconomic disadvantage. Patients with comorbid RDs had higher rates of cardiometabolic comorbidities including hypertension, DM, obesity, and hyperlipidemia, but lower rates of smoking (Table 2). RDs patients had higher rates of heart failure, atrial fibrillation, COPD and renal disease compared to those without – resulting in a significantly higher Charlson's comorbidity index (CCI), with almost 70 % of patients with ASCVD and comorbid RDs compared to 8.4 % of non-RD patients with ASCVD having a score of 4 or more indicating only a 53 % 10-year survival (Table 2).

**Table 1:** Baseline characteristics of the study population.

	No RD	RD				
	Overall	Overall	*p*-value	Female	Male	*p*-value
**Sample**	*N* = 105,735	*N* = 7286		*N* = 5168	*N* = 2118	
**Age Category**			0.001			<0.001
<40 yrs	2001 (1.9 %)	134 (1.8 %)		111 (2.1 %)	23 (1. 1 %)	
40–64yrs	30,446 (28.8 %)	1955 (26. 8 %)		1440 (27.9 %)	515 (24.3 %)	
≥ 65 yrs	73,288 (69.3 %)	5197 (71. 3 %)		3617 (70.0 %)	1580 (74.6 %)	
**Gender**			<0.001			<0.001
Female	45,685 (43.2 %)	5168 (70. 9 %)				
Male	60,050 (56.8 %)	2118 (29. 1 %)				
**Race/ethnicity**			<0.001			<0.001
Non-Hispanic White Americans	67,225 (63.6 %)	4555 (62. 5 %)		3077 (59.5 %)	1478 (69.8 %)	
Non-Hispanic Black Americans	15,656 (14.8 %)	1404 (19. 3 %)		1157 (22.4 %)	247 (11.7 %)	
Non-Hispanic Asian Americans	6301 (6.0 %)	316 (4.3 %)		202 (3.9 %)	114 (5. 4 %)	
Hispanic Americans	12,495 (11.8 %)	869 (11. 9 %)		640 (12.4 %)	229 (10. 8 %)	
Other	2185 (2.1 %)	78 (1.1 %)		52 (1.0 %)	26 (1.2 %)	
Unknown Ethnicity	1873 (1.8 %)	64 (0.9 %)		40 (0.8 %)	24 (1. 1 %)	
**ADI Quintiles**			0.003			<0.001
Quintile I (Least Deprived)	19,844 (18.8 %)	1284 (17. 6 %)		867 (16.8 %)	417 (19. 7 %)	
Quintile II	28,532 (27.0 %)	1895 (26. 0 %)		1313 (25.4 %)	582 (27.5 %)	
Quintile III	25,126 (23.8 %)	1806 (24. 8 %)		1264 (24.5 %)	542 (25.6 %)	
Quintile IV	19,717 (18.6 %)	1437 (19. 7 %)		1085 (21.0 %)	352 (16.6 %)	
Quintile V (Most Deprived)	11,744 (11.1 %)	825 (11. 3 %)		611 (11.8 %)	214 (10. 1 %)	
ADI N/A	772 (0.7 %)	39 (0. 5 %)		28 (0.5 %)	11 (0. 5 %)

**Table 2:** Patients with ASCVD with and without comorbid RDs.

	No RD	RD				
	Overall	Overall	*p*-value	Female	Male	*p*-value
**Sample**	*N* = 105,735	*N* = 7286		*N* = 5168	*N* = 2118	
**Lipid Parameters (mean ± SD)**						
LDL-C, mg/dL	85.8 (37.5)	85.9 (37.2)	0.83	89.7 (37.8)	76.6 (33.9)	<0.001
HDL-C, mg/dL	50.3 (16.7)	54.1 (17.9)	<0.001	57.4 (18.2)	46.0 (14.3)	<0.001
Triglycerides, mg/dL	127.6 (95.3)	124.6 (88.3)	0.02	123.9 (91.0)	126.2 (81.6)	0.36
**Risk factors**						
Hypertension	94,011 (88.9 %)	6657 (91. 4 %)	<0.001	4660 (90.2 %)	1997 (94.3 %)	<0.001
Diabetes mellitus	45,706 (43.2 %)	3252 (44. 6 %)	0.019	2185 (42.3 %)	1067 (50.4 %)	<0.001
Obesity	38,289 (36.2 %)	2798 (38. 4 %)	<0.001	2050 (39.7 %)	748 (3 5 %)	
Dyslipidemia	58,431 (55.3 %)	4396 (60. 3 %)	<0.001	3115 (60.3 %)	1281 (60.5 %)	0.87
Current smoking	9353 (8.8 %)	527 (7.2 %)	<0.001	358 (6.9 %)	169 (8. 0 %)	0.12
**Charlson's comorbidity index**						
0 CCI	6934 (6.6 %)	42 (0.6 %)	<0.001	25 (0.5 %)	17 (0. 8 %)	<0.001
1–3 CCI	53,404 (50.5 %)	2201 (30. 2 %)	<0.001	1633 (31.6 %)	568 (26.8 %)	<0.001
>=4 CI	45,397 (42.9 %)	5043 (69. 2 %)	<0.001	3510 (67.9 %)	1533 (72.4 %)	<0.001
**Comorbidities**						
Renal disease	32,018 (30.3 %)	2809 (38. 6 %)	<0.001	1885 (36.5 %)	924 (43.6 %)	<0.001
COPD	31,148 (29.5 %)	3495 (48. 0 %)	<0.001	2562 (49.6 %)	933 (44.1 %)	<0.001
Heart failure	31,020 (29.3 %)	2780 (38. 2 %)	<0.001	1883 (36.4 %)	897 (42.4 %)	<0.001
Atrial fibrillation	26,461 (25.0 %)	2087 (28. 6 %)	<0.001	1335 (25.8 %)	752 (35.5 %)	<0.001
**Lipid lowering medication**						
Any statin	75,861 (71.7 %)	4607 (63. 2 %)	<0.001	3081 (59.6 %)	1526 (72.0 %)	<0.001
High intensity statin	40,424 (38.2 %)	2212 (30. 4 %)	<0.001	1356 (26.2 %)	856 (40.4 %)	<0.001
PCSK9i	2423 (2.3 %)	218 (3.0 %)	<0.001	148 (2.9 %)	70 (3. 3 %)	0.32
Ezetimibe	11,354 (10.7 %)	853 (11. 7 %)	0.010	585 (11.3 %)	268 (12. 7 %)	0.11
Bempedoic acid	225 (0.2 %)	19 (0. 3 %)	0.39	12 (0.2 %)	7 (0. 3 %)	0.46

The most common comorbid RDs was Rheumatoid arthritis, followed by Systemic Lupus erythematosus and Sjogren syndrome (Table 3).

**Table 3:** Patients with comorbid ASCVD and RDs- distribution of different RDs by gender.

Rheumatoid Diseases	Overall	Female	Male	*p*-value
	*N* = 7286	*N* = 5168	*N* = 2118	
Rheumatoid Arthritis (RA)	4598 (63.1 % )	3209 (69. 8 %)	1389 (30.2 % )	0.005
Spondylo Psoriatic Arthritis (SPA)	681 (9.3 %)	345 (50. 6 %)	336 (49.4 %)	<0.001
Systemic Lupus Erythematosus (SLE)	1438 (19.7 %)	1203 (83.6 %)	235 (16.4 %)	<0.001
Systemic Sclerosis (SS)	314 (4.3 %)	266 (84. 7 %)	48 (15.3 %)	<0.001
Sjogren's Syndrome (SJS)	1201 (16.5 %)	1038 (86.4 %)	163 (13.6 %)	<0.001
Mixed Connective Tiss9ue Disease (MCTD)	175 (2.4 %)	146 (83. 4 %)	29 (16.6 %)	<0.001
Inflammatory Myopathy (IM)	225 (3.1 %)	142 (63. 1 %)	83 (36.7 %)	0.009

### 3.1 Utilization of statin

**Fig. 2:**
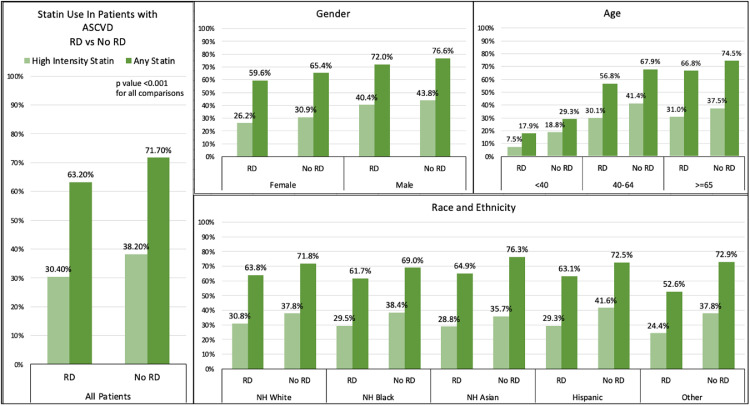
Utilization of Statins in Patients with Atherosclerotic Cardiovascular Disease across age strata, gender and racial/ethnic subgroups, stratified by RD status. RDs include Rheumatoid arthritis (RA), Juvenile RA, Psoriatic Arthritis and Ankylosing Spondylitis, Systemic Lupus Erythematosus (SLE), Systemic Sclerosis (SS), Sjogren Syndrome (SjS), Mixed connective tissue disease (MCTD), Inflammatory Myopathies (IM).

**Fig. 3:**
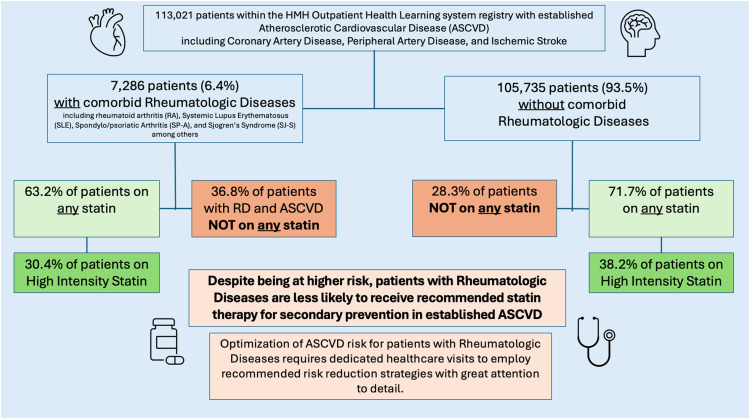
Central figure.

Overall, 71.2 % of ASCVD patients (80,468) were prescribed a statin (“any statin” prescription); the percentage of patients on any statin was significantly lower in the population of patients with ASCVD and RDs compared to the non-RD group (63.2 % vs. 71.7 %; *P* < 0.001) – (Figs. 2 and 3). These trends remained consistent across gender, age, and ethno-racial demographics and valid for high intensity statin prescriptions as well.

Statin prescriptions increased with age in both groups, with again overall less frequent prescriptions for patients with comorbid RDs in all age groups – (Fig. 2). The majority of patients aged > 65 were receiving a statin (74.5 % vs 66.8 %, no RD vs RDs cohort, *p* < 0.01) but a significant lower percentage were receiving high intensity statins (37.5 % vs 31.0 %, no RD vs RDs cohort, *p* < 0.01). In the younger age strata (age < 40) 29.3 % of ASCVD patients with no RD vs 17.9 % of patients with comorbid RDs were prescribed a statin. As observed for “Any statin”, prescription of high intensity statin was lower in ASCVD patients with comorbid RDs - across age, gender and racial/ethnics groups. In the younger age strata (age < 40) 18.8 % of ASCVD patients with no RD vs only 7.5 % of patients with comorbid RDs were prescribed a high intensity statin. Women with comorbid RD were less likely than their RD-free counterparts to be prescribed any statin (59.6% vs 65.4 %, *p* < 0.01) or high intensity statins (26.2 % vs 30.9 %) (Fig. 2).

Multivariate analysis showed how patients with comorbid RDs were on average 20 % less likely to be prescribed any statin and high intensity statin compared to patients with ASCVD without comorbid RDs in both a baseline model that adjusted only for demographics (M1) and a second model that took into consideration further risk factors/ comorbidities and use of non-statin lipid lowering treatments (M2) [M1- OR for any statin 0.77 (0.74–0.82) and M2- 0.79 (0.75–0.83); OR for high intensity statin (M1- 0.82(0.77–0.86) and M2- 0.80 (0.76–0.85), *p* < 0.001), (Table 5). Odds of low-moderate intensity statin were slightly lower for patients with comorbid RD in the initial model, but differences were no longer statistically significant in the fully adjusted model. Additionally, we considered the possibility that patients with RDs may have been treated with non-statin lipid lowering therapies. Our data showed that there was a statistically significant increase in use of PCSK9i (0.7 % increase) and Ezetimibe (1 % increase) in RD patients compared to non-RD patients (Table 2). However, these increases are minimal and are unlikely to be clinically significant to account for reduced statin utilization in this population.

Lastly, we evaluated statin use in a subgroup of the most common rheumatoid diseases including RA, SLE, SPA, and SJS. Statistical analysis was conducted on specific RD subgroups in comparison to those without the particular RD (ie. patients with RA versus all those without RA). The prescription of “any statin” was significantly lower in all 4 groups (Tables 3 and 4). No significant differences were observed in prescribing low-moderate intensity statin. However, patients with RA, SLE, SPA, and SJS were less likely to be prescribed high-intensity statin (Table 3).

**Table 4:** Statin use in patients with specific RDs.

Rheumatoid Diseases	Overall	Any Statin	High intensity statin
Rheumatoid Arthritis (RA)	4598	3014 (65.6 % )	1460 (31.8 %)
No RA	108,423	77,454 (71.4 %)	41,176 (38. 0 %)
*p*-value		<0.001	<0.01
Systemic Lupus Erythematosus (SLE)	1438	842 (58.6 % )	418 (29. 1 %)
No SLE	111,583	79,626 (71.4 %)	42,218 (37. 8 %)
*p*-value		<0.001	<0.001
SPA	681	446 (65.5 %)	217 (31. 9 %)
No SPA	112,340	80,022 (71.2 %)	42,419 (37. 8 %)
*p*-value		<0.001	0.002
Sjogren's Syndrome (SJS)	1201	711 (59.2 % )	303 (25. 2 %)
No SJS	111,820	79,757 (71.3 %)	42,333 (37. 9 %)
*p*-value		<0.001	<0.001

**Table 5:** Odds of Statin Utilization in ASCVD patient with vs wo Comorbid RDs.

		Any Statin		Low-moderate Intensity Statin		High-Intensity Statin	
	No RD	RD (OR 95 % CI)	*p* value	RD (OR 95 % CI)	*p* value	RD (OR 95 % CI)	*p* value
ASCVD							
Model 1	1 (Ref)	0.77 (0.74–0.82)	<0.001	0.94 (0.89–0.98)	0.013	0.82 (0.77–0.86)	<0.001
Model 2	1 (Ref)	0.79 (0.75–0.83)	<0.001	0.98 (0.93–1.03)	0.339	0.80 (0.76–0.85)	<0.001

Model 1 is adjusted for demographics including age and race/ethnicity

Model 2 is adjusted for model 1 + insurance status, chronic kidney disease, cerebrovascular disease, diabetes mellitus, obesity, smoking, hypertension, heart failure, Charlson-comorbidity index, non-statin lipid lowering meds

ASCVD = atherosclerotic cardiovascular disease; CAD = coronary artery disease; PAD= peripheral artery disease.

### 3.2 Gender disparities

Women with ASCVD both with and without RDs were overall less likely to be prescribed any statin or high intensity statin than their male counterparts. These trends are shown in Tables 6 and 7.

**Table 6:** Secondary prevention statin utilization rates in RDs and non RDs cohorts by gender.

		Any Statin		Low-moderate Intensity Statin			High-Intensity Statin		
	No RD	RD	*p* value RD vs No RD	No RD	RD	*p* value RD vs No RD	No RD	RD	*p* value RD vs No RD
Female	65.4 %	59. 6 %	<0.001	35.3 %	33. 8 %	<0.001	30.9 %	26. 2 %	<0.001
Male	76.6 %	72. 0 %	<0.001	33.9 %	32. 7 %	<0.001	43.8 %	40. 4 %	<0.001
*p* value Female vs Male	<0.001	<0.001		<0.001	<0.001		<0.001	<0.001

**Table 7:** Odds of statin utilization in women vs men with ASCVD and comorbid RDs.

		Any Statin		Low-moderate Intensity Statin		High-Intensity Statin	
	Men	Women (OR 95 % CI)	*p* value	Women (OR 95 % CI)	*p* value	Women (OR 95 % CI)	*p* value
ASCVD							
Model 1	1 (Ref)	0.58 (0.52–0.65)	<0.001	1.07 (0.96–1.19)	0.206	0.53 (0.47−0.59)	<0.001
Model 2	1 (Ref)	0.66 (0.59–0.74)	<0.001	1.11 (0.99–1.24)	0.061	0.58 (0.52–0.65)	<0.001

Model 1 is adjusted for demographics including age and race/ethnicity.

Model 2 is adjusted for model 1 + insurance status, chronic kidney disease, cerebrovascular disease, diabetes mellitus, obesity, smoking, hypertension, heart failure, Charlson-comorbidity index, non-statin lipid lowering meds.

ASCVD, atherosclerotic cardiovascular disease; CAD, coronary artery disease; PAD, peripheral artery disease.

In multivariate models in patients with RDs, female gender was associated with a significant lower likelihood of any statin prescription and high intensity statin prescription, in both in the baseline (M1) and in the fully adjusted models (M2) (OR for any statin M1- 0.58 (0.52–0.65) and M2- 0.66 (0.59–0.74), *p* < 0.001; OR for high intensity statin M1- 0.53 (0.47 −0.59) and M2- 0.58 (0.52–0.65), *p* < 0.001). (Table 6). Notably female gender was not associated with lower odds of receiving a low-moderate statin prescription in either model.

## 4 Discussion

This cross-sectional study of 113,021 ASCVD patients from a single center institution highlights significant disparities in statin utilization among patients with and without comorbid RDs. Furthermore, gender disparities were evident in statin utilization among patients with ASCVD as well as in the group of patients with ASCVD and comorbid RDs. Co-morbid RDs were associated with lower overall statin utilization and lower utilization across age and ethnic subgroups. Women with RDs were almost 50 % less likely to be on high intensity statin compared to their male counterpart, however low-moderate intensity statin use were slightly higher among women compared to men both with and without comorbid RDs (Table 6).

Statin treatment in secondary prevention for ASCVD is supported by high level evidence and endorsed by multiple guidelines and societies [5,12,13]. Despite this, statin use remains widely suboptimal in the US population at large, with almost 40 % of adult patients with an established prior ASCVD event [corresponding approximately to 9.5 million Americans] not on statin treatment [14]. Ethnic and gender disparities have been highlighted, with younger, female patients being less likely to be actively treated with statins despite clear indications [[14], [15], [16]]. In the current analysis- rates of utilization of statins for secondary ASCVD prevention were higher than those reported in some of these recent studies, but are still far from ideal. Moreover, statin utilization was specifically lower in patients with concomitant diagnoses of rheumatologic disorders. To the best of our knowledge this is the first study that explored secondary ASCVD prevention among patients with RDs at large, including different RD subtypes, and in a large contemporary patient population utilizing real world EMR based data from over 100,000 patients in a large integrated health system. The missed opportunities for use of guideline-directed medical therapies in the RDs population with concomitant established ASCVD is concerning given the higher CV risk that these patients face. Not only are these patients at higher risk of developing ASCVD, but the lower utilization of proven risk reduction therapies among those with established ASCVD is a stark call to action.

A plethora of data have linked chronic inflammation to the development of atherosclerosis and ASCVD, and inflammation is recognized as a potential target for secondary prevention to further reduce risk reduction in patients with established ASCVD [17,18]. Chronic inflammation is a common feature among those with RDs, and together with concomitant traditional risk factors, explain the enhanced CVD risk in patients with RDs [[19], [20], [21], [22]]. Numerous studies have demonstrated the beneficial effects of statins in this population including reductions in mortality, ASCVD event rates, and in some studies inflammation and disease activity- highlighting the important role of statin therapy in providing both anti-inflammatory and anti-atherogenic effects [9,23,24]. Current International recommendation suggest relying on the same approach for ASCVD assessment and treatment for patients with RDs as for the general population [7,8]. To a minimum, then, patients with established ASCVD and comorbid RDs should be on high intensity statin. Contrary to current knowledge and clinical guidelines, our data suggest considerable gaps in statin utilization. The lack of focus on aggressive ASCVD management in the RD population is not new. Multiple studies have highlighted in the past the underdiagnosis and undertreatment of traditional cardiovascular risk factors – hypertension, dyslipidemia- always however limiting their focus on specific RDs [25,26]^,^ and attempted to identify the major driver of such gaps in care. Lack of expertise by the involved providers – be it in primary care or rheumatology– as well as lack of time were often highlighted as critical factors in the under-utilization of statins [22,27].

Our data expand on this field, highlighting that not only CV risk factors are underdiagnosed, but that treatment of established ASCVD with evidence based, class I indicated medical options is far from ideal across the spectrum of RDs in a contemporary large US population. In our study hyperlipidemia was more frequent in the RDs population compared to the ASCVD cohort, but average LDL values were comparable among the two groups with an average LDL below 100 mg/dl. The lipid paradox in chronic inflammation might partially explain these findings and may contribute to the lack of aggressive lipid lowering approaches in patients with RDs. The effects of chronic inflammation on lipid levels are well described, with uncontrolled inflammation resulting in lower total and LDL cholesterol [28]. The higher inflammatory burden present in patients with ASCVD and comorbid RDs might explain the similar LDL levels in this population despite lower statin use. At the same time, a normal LDL level in the lipid panel might not have prompted the clinician to start a statin prescription, or up-titrate to a high intensity statin, which remains indicated for the secondary prevention of ASCVD with LDL targets of <70 or <55 mg/dl, well below the normalcy threshold. Such nuances however might escape busy primary care providers or rheumatologists, partially contributing to gaps in care.

Finally in our study we observed considerable disparities in utilization patterns based on gender, both in the non-RDs and the RDs population, confirming a trend that has been described previously, with female patients being less likely to be prescribed statins for secondary prevention of ASCVD in different populations [15,[29], [30], [31]]. However, this gap appears particularly relevant in patients with established ASCVD and comorbid RDs. RDs are indeed as a whole more frequent in female patients [26], as reflected in our study population. In this prevalently female cohort appropriately addressing gender disparities might have a significant impact on outcomes, especially when considering the pleiotropic effects of statins and their impact on inflammation [9,23]. Although this is the first study to our knowledge to investigate gender disparities in comorbid ASCVD and RDs, reasons behind gender disparities in statin use at large have been investigated in prior studies and are multifactorial, encompassing historical underrepresentation of women in clinical trials [32], lack of awareness of CVD as a major health concern in women, higher reported side effects/intolerance [33,34] to statin treatment, higher hesitancy [30], and physician inertia. Women are less likely to initiate and continue statin treatment [30] and are less inclined to follow a physician recommendation to start statins compared to male patients [35]. This consideration can be applied to female patients with ASCVD and comorbid RDs. Concerns for medication side effects, most prominently myalgias, which are already more common in female patients [36] might be particularly relevant, as patients might be weary of the possibility of worsening baseline pain related to their disease. Regarding prescriber's inertia, concern for harm might play a significant role given the often young age of patients in this cohort. Many women with RDs diagnosis are of childbearing age, and the use of statins as lipid lowering agents is contraindicated during or while planning pregnancy unless specific high-risk criteria are met. However, it is worth reminding that the majority of these patients are actively being treated for their underlying RDs with chronic medications that are equally contraindicated during pregnancy and that require careful management of family planning. The physician should thus not be deterred from using effective evidence-based medications in this setting, provided the appropriate conversation and risk mitigation strategies are in place. Additionally, statin intolerance may also contribute to lower utilization of statins as lipid lowering therapy in this population, which may in part account for the lower utilization of high intensity statins in this patient population. However, the use of non-statin lipid lowering therapies were not significantly increased in the RDs vs non RDs cohorts, suggesting that statin intolerance is not the main barrier to statin utilization in this patient population.

These complexities in lipid management in patients with ASCVD and comorbid RDs underline the need for a multidisciplinary approach in which different specialties can come together, each providing the specific expertise required in the management of these high-risk patients. Furthermore, the immune dysregulation between the RDs may differ and relative CV risk may vary among the RDs and one challenge moving forward will be to understand whether different established therapeutic biologics for each of these RDs may be more or less beneficial once a patient develops ASCVD.

A few limitations of the present analysis need to be highlighted. This cross-sectional study depicts the experience of a single center, and as such, its applicability to the US population at large is limited. However, we were able to collect data on a large sample population and provide insight on a wide spectrum of RDs, with data on a contemporary US cohort that was, to the best of our knowledge, currently lacking. The inclusion of different RDs does raise the question of different underlying inflammatory involvement and burden, which might differentially impact the lipid profile of our patients. Yet statin indication in ASCVD secondary prevention is not affected by lipid levels.

Our selection criteria for inclusion in the study was based on the presence of appropriate diagnostic codes in the patient charts, as selected by experts in Rheumatic disease. This methodology, although validated for large EHR analysis, is prone to error and biases. We used the presence of any appropriate ICD at any time in the patient's EHR (Electronic Health Record), hence potentially decreasing the specificity of our analysis but casting as wide as possible a net to describe the magnitude of the issue in a large patient sample. Additionally, due the nature of this EHR based analysis, we are unable to report on patient adherence patterns or statin intolerance rates in these patient populations. These questions will require further research in this patient population. Finally, we selected a diverse group pf RDs but still focused on select autoimmune conditions that have previously been robustly associated with CVD risks. In doing so, we excluded conditions such as vasculitides, gout and osteoarthritis; future studies might shed further light on the association between these RDs and ASCVD.

## 5 Conclusion

The presence of co-morbid RDs was associated with lower utilization of guideline recommended secondary prevention statins in patients with ASCVD in this large contemporary single-center cross-sectional study. Female gender was associated with further reduction in statin utilization in this cohort.

Multidisciplinary team approaches and increased awareness among clinicians are needed to amplify evidence-based preventive strategies in this high-risk demographic.

## CRediT authorship contribution statement

**Eleonora Avenatti:** Writing – review & editing, Writing – original draft, Visualization, Validation, Supervision, Software, Resources, Project administration, Methodology, Investigation, Formal analysis, Data curation, Conceptualization. **Helene DiGregorio:** Writing – review & editing, Writing – original draft, Visualization, Validation, Supervision, Resources, Project administration, Methodology, Investigation, Formal analysis, Data curation, Conceptualization. **Elia El Hajj:** Writing – review & editing, Writing – original draft, Visualization, Formal analysis. **Rakesh Gullapelli:** Validation, Software, Methodology, Formal analysis, Data curation, Conceptualization. **Kenneth Williams:** Writing – review & editing, Visualization, Validation, Investigation, Formal analysis, Data curation. **Izza Shahid:** Supervision, Software, Resources, Project administration, Formal analysis, Data curation. **Budhaditya Bose:** Software, Resources, Investigation, Formal analysis, Data curation. **Kobina Hagan:** Validation, Software, Resources, Project administration, Investigation, Formal analysis. **Juan C Nicolas:** Software, Resources, Investigation, Formal analysis. **Shubham Lahan:** Software, Resources, Methodology, Formal analysis, Data curation. **Nwabunie Nwana:** Software, Resources, Project administration, Formal analysis, Data curation. **Sara Ayaz Butt:** Supervision, Software, Methodology, Investigation, Formal analysis, Data curation. **Kanika Monga:** Writing – review & editing, Validation, Supervision, Methodology, Formal analysis, Conceptualization. **Lily Anne Romero Karam:** Writing – review & editing, Visualization, Validation, Methodology, Investigation, Formal analysis, Conceptualization. **Myriam Guevara:** Writing – review & editing, Writing – original draft, Visualization, Validation, Methodology, Investigation, Formal analysis, Conceptualization. **Zulqarnain Javed:** Writing – review & editing, Visualization, Validation, Supervision, Software, Resources, Project administration, Methodology, Investigation, Funding acquisition, Formal analysis, Data curation, Conceptualization. **Brittany Weber:** Writing – review & editing, Visualization, Supervision, Methodology, Investigation, Formal analysis. **Sadeer Al-Kindi:** Writing – review & editing, Validation, Supervision, Software, Resources, Methodology, Investigation, Funding acquisition, Formal analysis, Data curation, Conceptualization. **Khurram Nasir:** Writing – review & editing, Writing – original draft, Visualization, Validation, Supervision, Software, Resources, Project administration, Methodology, Investigation, Funding acquisition, Formal analysis, Data curation, Conceptualization.

## Declaration of competing interest

The authors declare that they have no conflicts of interest relevant to the content of this manuscript.

## Appendix Supplementary materials

Image, application 1
