# Supplementary material for: Disparities in statin use in patients with ASCVD with vs without rheumatologic diseases in a large integrated healthcare system: Houston methodist CVD learning health system registry
Source: Am J Prev Cardiol. 2025 Mar 28;22:100959. doi: 10.1016/j.ajpc.2025.100959 (PMC12023777; doi:10.1016/j.ajpc.2025.100959)
Supplement: Supplementary file 1 [file mmc1.docx]

**Supplementary Appendix**

**Supplemental Table 1-3 ICD 10 codes used to identify Rheumatologic diseases**

**1a- Rheumatoid Arthritis**

**1b- Juvenile Rheumatoid Arthritis**

**2a- Spondyloarthritis (Ankylosing Spondylitis, psoriatic arthritis)**

**2b- Systemic Lupus Erythematosus**

**2c- Mixed Connective Tissue Disease**

**3a- Systemic Sclerosis**

**3b- Inflammatory Myopathy**

**3c- Sjogren Syndrome**

**Supplementary Tables 1-3 : ICD 10 codes used to identify Rheumatological diseases in the study population**

1a - Rheumatoid Arthritis codes

| *[M05](https://www.icd10data.com/ICD10CM/Codes/M00-M99/M05-M14/M05-/M05)* | **Rheumatoid arthritis with rheumatoid factor** |
| --- | --- |
| [*M05.0*](https://www.icd10data.com/ICD10CM/Codes/M00-M99/M05-M14/M05-/M05.0) | Felty's syndrome |
| [*M05.00*](https://www.icd10data.com/ICD10CM/Codes/M00-M99/M05-M14/M05-/M05.00) | …… unspecified site |
| [*M05.01*](https://www.icd10data.com/ICD10CM/Codes/M00-M99/M05-M14/M05-/M05.01) | Felty's syndrome, shoulder |
| [*M05.011*](https://www.icd10data.com/ICD10CM/Codes/M00-M99/M05-M14/M05-/M05.011) | Felty's syndrome, right shoulder |
| [*M05.012*](https://www.icd10data.com/ICD10CM/Codes/M00-M99/M05-M14/M05-/M05.012) | Felty's syndrome, left shoulder |
| [*M05.019*](https://www.icd10data.com/ICD10CM/Codes/M00-M99/M05-M14/M05-/M05.019) | Felty's syndrome, unspecified shoulder |
| [*M05.02*](https://www.icd10data.com/ICD10CM/Codes/M00-M99/M05-M14/M05-/M05.02) | Felty's syndrome, elbow |
| [*M05.021*](https://www.icd10data.com/ICD10CM/Codes/M00-M99/M05-M14/M05-/M05.021) | Felty's syndrome, right elbow |
| [*M05.022*](https://www.icd10data.com/ICD10CM/Codes/M00-M99/M05-M14/M05-/M05.022) | Felty's syndrome, left elbow |
| [*M05.029*](https://www.icd10data.com/ICD10CM/Codes/M00-M99/M05-M14/M05-/M05.029) | Felty's syndrome, unspecified elbow |
| [*M05.03*](https://www.icd10data.com/ICD10CM/Codes/M00-M99/M05-M14/M05-/M05.03) | Felty's syndrome, wrist |
| [*M05.031*](https://www.icd10data.com/ICD10CM/Codes/M00-M99/M05-M14/M05-/M05.031) | Felty's syndrome, right wrist |
| [*M05.032*](https://www.icd10data.com/ICD10CM/Codes/M00-M99/M05-M14/M05-/M05.032) | Felty's syndrome, left wrist |
| [*M05.039*](https://www.icd10data.com/ICD10CM/Codes/M00-M99/M05-M14/M05-/M05.039) | Felty's syndrome, unspecified wrist |
| [*M05.04*](https://www.icd10data.com/ICD10CM/Codes/M00-M99/M05-M14/M05-/M05.04) | Felty's syndrome, hand |
| [*M05.041*](https://www.icd10data.com/ICD10CM/Codes/M00-M99/M05-M14/M05-/M05.041) | Felty's syndrome, right hand |
| [*M05.042*](https://www.icd10data.com/ICD10CM/Codes/M00-M99/M05-M14/M05-/M05.042) | Felty's syndrome, left hand |
| [*M05.049*](https://www.icd10data.com/ICD10CM/Codes/M00-M99/M05-M14/M05-/M05.049) | Felty's syndrome, unspecified hand |
| [*M05.05*](https://www.icd10data.com/ICD10CM/Codes/M00-M99/M05-M14/M05-/M05.05) | Felty's syndrome, hip |
| [*M05.051*](https://www.icd10data.com/ICD10CM/Codes/M00-M99/M05-M14/M05-/M05.051) | Felty's syndrome, right hip |
| [*M05.052*](https://www.icd10data.com/ICD10CM/Codes/M00-M99/M05-M14/M05-/M05.052) | Felty's syndrome, left hip |
| [*M05.059*](https://www.icd10data.com/ICD10CM/Codes/M00-M99/M05-M14/M05-/M05.059) | Felty's syndrome, unspecified hip |
| [*M05.06*](https://www.icd10data.com/ICD10CM/Codes/M00-M99/M05-M14/M05-/M05.06) | Felty's syndrome, knee |
| [*M05.061*](https://www.icd10data.com/ICD10CM/Codes/M00-M99/M05-M14/M05-/M05.061) | Felty's syndrome, right knee |
| [*M05.062*](https://www.icd10data.com/ICD10CM/Codes/M00-M99/M05-M14/M05-/M05.062) | Felty's syndrome, left knee |
| [*M05.069*](https://www.icd10data.com/ICD10CM/Codes/M00-M99/M05-M14/M05-/M05.069) | Felty's syndrome, unspecified knee |
| [*M05.07*](https://www.icd10data.com/ICD10CM/Codes/M00-M99/M05-M14/M05-/M05.07) | Felty's syndrome, ankle and foot |
| [*M05.071*](https://www.icd10data.com/ICD10CM/Codes/M00-M99/M05-M14/M05-/M05.071) | Felty's syndrome, right ankle and foot |
| [*M05.072*](https://www.icd10data.com/ICD10CM/Codes/M00-M99/M05-M14/M05-/M05.072) | Felty's syndrome, left ankle and foot |
| [*M05.079*](https://www.icd10data.com/ICD10CM/Codes/M00-M99/M05-M14/M05-/M05.079) | Felty's syndrome, unspecified ankle and foot |
| [*M05.09*](https://www.icd10data.com/ICD10CM/Codes/M00-M99/M05-M14/M05-/M05.09) | …… multiple sites |
| [*M05.1*](https://www.icd10data.com/ICD10CM/Codes/M00-M99/M05-M14/M05-/M05.1) | Rheumatoid lung disease with rheumatoid arthritis |
| [*M05.10*](https://www.icd10data.com/ICD10CM/Codes/M00-M99/M05-M14/M05-/M05.10) | …… of unspecified site |
| [*M05.11*](https://www.icd10data.com/ICD10CM/Codes/M00-M99/M05-M14/M05-/M05.11) | Rheumatoid lung disease with rheumatoid arthritis of shoulder |
| [*M05.111*](https://www.icd10data.com/ICD10CM/Codes/M00-M99/M05-M14/M05-/M05.111) | Rheumatoid lung disease with rheumatoid arthritis of right shoulder |
| [*M05.112*](https://www.icd10data.com/ICD10CM/Codes/M00-M99/M05-M14/M05-/M05.112) | Rheumatoid lung disease with rheumatoid arthritis of left shoulder |
| [*M05.119*](https://www.icd10data.com/ICD10CM/Codes/M00-M99/M05-M14/M05-/M05.119) | Rheumatoid lung disease with rheumatoid arthritis of unspecified shoulder |
| [*M05.12*](https://www.icd10data.com/ICD10CM/Codes/M00-M99/M05-M14/M05-/M05.12) | Rheumatoid lung disease with rheumatoid arthritis of elbow |
| [*M05.121*](https://www.icd10data.com/ICD10CM/Codes/M00-M99/M05-M14/M05-/M05.121) | Rheumatoid lung disease with rheumatoid arthritis of right elbow |
| [*M05.122*](https://www.icd10data.com/ICD10CM/Codes/M00-M99/M05-M14/M05-/M05.122) | Rheumatoid lung disease with rheumatoid arthritis of left elbow |
| [*M05.129*](https://www.icd10data.com/ICD10CM/Codes/M00-M99/M05-M14/M05-/M05.129) | Rheumatoid lung disease with rheumatoid arthritis of unspecified elbow |
| [*M05.13*](https://www.icd10data.com/ICD10CM/Codes/M00-M99/M05-M14/M05-/M05.13) | Rheumatoid lung disease with rheumatoid arthritis of wrist |
| [*M05.131*](https://www.icd10data.com/ICD10CM/Codes/M00-M99/M05-M14/M05-/M05.131) | Rheumatoid lung disease with rheumatoid arthritis of right wrist |
| [*M05.132*](https://www.icd10data.com/ICD10CM/Codes/M00-M99/M05-M14/M05-/M05.132) | Rheumatoid lung disease with rheumatoid arthritis of left wrist |
| [*M05.139*](https://www.icd10data.com/ICD10CM/Codes/M00-M99/M05-M14/M05-/M05.139) | Rheumatoid lung disease with rheumatoid arthritis of unspecified wrist |
| [*M05.14*](https://www.icd10data.com/ICD10CM/Codes/M00-M99/M05-M14/M05-/M05.14) | Rheumatoid lung disease with rheumatoid arthritis of hand |
| [*M05.141*](https://www.icd10data.com/ICD10CM/Codes/M00-M99/M05-M14/M05-/M05.141) | Rheumatoid lung disease with rheumatoid arthritis of right hand |
| [*M05.142*](https://www.icd10data.com/ICD10CM/Codes/M00-M99/M05-M14/M05-/M05.142) | Rheumatoid lung disease with rheumatoid arthritis of left hand |
| [*M05.149*](https://www.icd10data.com/ICD10CM/Codes/M00-M99/M05-M14/M05-/M05.149) | Rheumatoid lung disease with rheumatoid arthritis of unspecified hand |
| [*M05.15*](https://www.icd10data.com/ICD10CM/Codes/M00-M99/M05-M14/M05-/M05.15) | Rheumatoid lung disease with rheumatoid arthritis of hip |
| [*M05.151*](https://www.icd10data.com/ICD10CM/Codes/M00-M99/M05-M14/M05-/M05.151) | Rheumatoid lung disease with rheumatoid arthritis of right hip |
| [*M05.152*](https://www.icd10data.com/ICD10CM/Codes/M00-M99/M05-M14/M05-/M05.152) | Rheumatoid lung disease with rheumatoid arthritis of left hip |
| [*M05.159*](https://www.icd10data.com/ICD10CM/Codes/M00-M99/M05-M14/M05-/M05.159) | Rheumatoid lung disease with rheumatoid arthritis of unspecified hip |
| [*M05.16*](https://www.icd10data.com/ICD10CM/Codes/M00-M99/M05-M14/M05-/M05.16) | Rheumatoid lung disease with rheumatoid arthritis of knee |
| [*M05.161*](https://www.icd10data.com/ICD10CM/Codes/M00-M99/M05-M14/M05-/M05.161) | Rheumatoid lung disease with rheumatoid arthritis of right knee |
| [*M05.162*](https://www.icd10data.com/ICD10CM/Codes/M00-M99/M05-M14/M05-/M05.162) | Rheumatoid lung disease with rheumatoid arthritis of left knee |
| [*M05.169*](https://www.icd10data.com/ICD10CM/Codes/M00-M99/M05-M14/M05-/M05.169) | Rheumatoid lung disease with rheumatoid arthritis of unspecified knee |
| [*M05.17*](https://www.icd10data.com/ICD10CM/Codes/M00-M99/M05-M14/M05-/M05.17) | Rheumatoid lung disease with rheumatoid arthritis of ankle and foot |
| [*M05.171*](https://www.icd10data.com/ICD10CM/Codes/M00-M99/M05-M14/M05-/M05.171) | Rheumatoid lung disease with rheumatoid arthritis of right ankle and foot |
| [*M05.172*](https://www.icd10data.com/ICD10CM/Codes/M00-M99/M05-M14/M05-/M05.172) | Rheumatoid lung disease with rheumatoid arthritis of left ankle and foot |
| [*M05.179*](https://www.icd10data.com/ICD10CM/Codes/M00-M99/M05-M14/M05-/M05.179) | Rheumatoid lung disease with rheumatoid arthritis of unspecified ankle and foot |
| [*M05.19*](https://www.icd10data.com/ICD10CM/Codes/M00-M99/M05-M14/M05-/M05.19) | …… of multiple sites |
| [*M05.2*](https://www.icd10data.com/ICD10CM/Codes/M00-M99/M05-M14/M05-/M05.2) | Rheumatoid vasculitis with rheumatoid arthritis |
| [*M05.20*](https://www.icd10data.com/ICD10CM/Codes/M00-M99/M05-M14/M05-/M05.20) | …… of unspecified site |
| [*M05.21*](https://www.icd10data.com/ICD10CM/Codes/M00-M99/M05-M14/M05-/M05.21) | Rheumatoid vasculitis with rheumatoid arthritis of shoulder |
| [*M05.211*](https://www.icd10data.com/ICD10CM/Codes/M00-M99/M05-M14/M05-/M05.211) | Rheumatoid vasculitis with rheumatoid arthritis of right shoulder |
| [*M05.212*](https://www.icd10data.com/ICD10CM/Codes/M00-M99/M05-M14/M05-/M05.212) | Rheumatoid vasculitis with rheumatoid arthritis of left shoulder |
| [*M05.219*](https://www.icd10data.com/ICD10CM/Codes/M00-M99/M05-M14/M05-/M05.219) | Rheumatoid vasculitis with rheumatoid arthritis of unspecified shoulder |
| [*M05.22*](https://www.icd10data.com/ICD10CM/Codes/M00-M99/M05-M14/M05-/M05.22) | Rheumatoid vasculitis with rheumatoid arthritis of elbow |
| [*M05.221*](https://www.icd10data.com/ICD10CM/Codes/M00-M99/M05-M14/M05-/M05.221) | Rheumatoid vasculitis with rheumatoid arthritis of right elbow |
| [*M05.222*](https://www.icd10data.com/ICD10CM/Codes/M00-M99/M05-M14/M05-/M05.222) | Rheumatoid vasculitis with rheumatoid arthritis of left elbow |
| [*M05.229*](https://www.icd10data.com/ICD10CM/Codes/M00-M99/M05-M14/M05-/M05.229) | Rheumatoid vasculitis with rheumatoid arthritis of unspecified elbow |
| [*M05.23*](https://www.icd10data.com/ICD10CM/Codes/M00-M99/M05-M14/M05-/M05.23) | Rheumatoid vasculitis with rheumatoid arthritis of wrist |
| [*M05.231*](https://www.icd10data.com/ICD10CM/Codes/M00-M99/M05-M14/M05-/M05.231) | Rheumatoid vasculitis with rheumatoid arthritis of right wrist |
| [*M05.232*](https://www.icd10data.com/ICD10CM/Codes/M00-M99/M05-M14/M05-/M05.232) | Rheumatoid vasculitis with rheumatoid arthritis of left wrist |
| [*M05.239*](https://www.icd10data.com/ICD10CM/Codes/M00-M99/M05-M14/M05-/M05.239) | Rheumatoid vasculitis with rheumatoid arthritis of unspecified wrist |
| [*M05.24*](https://www.icd10data.com/ICD10CM/Codes/M00-M99/M05-M14/M05-/M05.24) | Rheumatoid vasculitis with rheumatoid arthritis of hand |
| [*M05.241*](https://www.icd10data.com/ICD10CM/Codes/M00-M99/M05-M14/M05-/M05.241) | Rheumatoid vasculitis with rheumatoid arthritis of right hand |
| [*M05.242*](https://www.icd10data.com/ICD10CM/Codes/M00-M99/M05-M14/M05-/M05.242) | Rheumatoid vasculitis with rheumatoid arthritis of left hand |
| [*M05.249*](https://www.icd10data.com/ICD10CM/Codes/M00-M99/M05-M14/M05-/M05.249) | Rheumatoid vasculitis with rheumatoid arthritis of unspecified hand |
| [*M05.25*](https://www.icd10data.com/ICD10CM/Codes/M00-M99/M05-M14/M05-/M05.25) | Rheumatoid vasculitis with rheumatoid arthritis of hip |
| [*M05.251*](https://www.icd10data.com/ICD10CM/Codes/M00-M99/M05-M14/M05-/M05.251) | Rheumatoid vasculitis with rheumatoid arthritis of right hip |
| [*M05.252*](https://www.icd10data.com/ICD10CM/Codes/M00-M99/M05-M14/M05-/M05.252) | Rheumatoid vasculitis with rheumatoid arthritis of left hip |
| [*M05.259*](https://www.icd10data.com/ICD10CM/Codes/M00-M99/M05-M14/M05-/M05.259) | Rheumatoid vasculitis with rheumatoid arthritis of unspecified hip |
| [*M05.26*](https://www.icd10data.com/ICD10CM/Codes/M00-M99/M05-M14/M05-/M05.26) | Rheumatoid vasculitis with rheumatoid arthritis of knee |
| [*M05.261*](https://www.icd10data.com/ICD10CM/Codes/M00-M99/M05-M14/M05-/M05.261) | Rheumatoid vasculitis with rheumatoid arthritis of right knee |
| [*M05.262*](https://www.icd10data.com/ICD10CM/Codes/M00-M99/M05-M14/M05-/M05.262) | Rheumatoid vasculitis with rheumatoid arthritis of left knee |
| [*M05.269*](https://www.icd10data.com/ICD10CM/Codes/M00-M99/M05-M14/M05-/M05.269) | Rheumatoid vasculitis with rheumatoid arthritis of unspecified knee |
| [*M05.27*](https://www.icd10data.com/ICD10CM/Codes/M00-M99/M05-M14/M05-/M05.27) | Rheumatoid vasculitis with rheumatoid arthritis of ankle and foot |
| [*M05.271*](https://www.icd10data.com/ICD10CM/Codes/M00-M99/M05-M14/M05-/M05.271) | Rheumatoid vasculitis with rheumatoid arthritis of right ankle and foot |
| [*M05.272*](https://www.icd10data.com/ICD10CM/Codes/M00-M99/M05-M14/M05-/M05.272) | Rheumatoid vasculitis with rheumatoid arthritis of left ankle and foot |
| [*M05.279*](https://www.icd10data.com/ICD10CM/Codes/M00-M99/M05-M14/M05-/M05.279) | Rheumatoid vasculitis with rheumatoid arthritis of unspecified ankle and foot |
| [*M05.29*](https://www.icd10data.com/ICD10CM/Codes/M00-M99/M05-M14/M05-/M05.29) | …… of multiple sites |
| [*M05.3*](https://www.icd10data.com/ICD10CM/Codes/M00-M99/M05-M14/M05-/M05.3) | Rheumatoid heart disease with rheumatoid arthritis |
| [*M05.30*](https://www.icd10data.com/ICD10CM/Codes/M00-M99/M05-M14/M05-/M05.30) | …… of unspecified site |
| [*M05.31*](https://www.icd10data.com/ICD10CM/Codes/M00-M99/M05-M14/M05-/M05.31) | Rheumatoid heart disease with rheumatoid arthritis of shoulder |
| [*M05.311*](https://www.icd10data.com/ICD10CM/Codes/M00-M99/M05-M14/M05-/M05.311) | Rheumatoid heart disease with rheumatoid arthritis of right shoulder |
| [*M05.312*](https://www.icd10data.com/ICD10CM/Codes/M00-M99/M05-M14/M05-/M05.312) | Rheumatoid heart disease with rheumatoid arthritis of left shoulder |
| [*M05.319*](https://www.icd10data.com/ICD10CM/Codes/M00-M99/M05-M14/M05-/M05.319) | Rheumatoid heart disease with rheumatoid arthritis of unspecified shoulder |
| [*M05.32*](https://www.icd10data.com/ICD10CM/Codes/M00-M99/M05-M14/M05-/M05.32) | Rheumatoid heart disease with rheumatoid arthritis of elbow |
| [*M05.321*](https://www.icd10data.com/ICD10CM/Codes/M00-M99/M05-M14/M05-/M05.321) | Rheumatoid heart disease with rheumatoid arthritis of right elbow |
| [*M05.322*](https://www.icd10data.com/ICD10CM/Codes/M00-M99/M05-M14/M05-/M05.322) | Rheumatoid heart disease with rheumatoid arthritis of left elbow |
| [*M05.329*](https://www.icd10data.com/ICD10CM/Codes/M00-M99/M05-M14/M05-/M05.329) | Rheumatoid heart disease with rheumatoid arthritis of unspecified elbow |
| [*M05.33*](https://www.icd10data.com/ICD10CM/Codes/M00-M99/M05-M14/M05-/M05.33) | Rheumatoid heart disease with rheumatoid arthritis of wrist |
| [*M05.331*](https://www.icd10data.com/ICD10CM/Codes/M00-M99/M05-M14/M05-/M05.331) | Rheumatoid heart disease with rheumatoid arthritis of right wrist |
| [*M05.332*](https://www.icd10data.com/ICD10CM/Codes/M00-M99/M05-M14/M05-/M05.332) | Rheumatoid heart disease with rheumatoid arthritis of left wrist |
| [*M05.339*](https://www.icd10data.com/ICD10CM/Codes/M00-M99/M05-M14/M05-/M05.339) | Rheumatoid heart disease with rheumatoid arthritis of unspecified wrist |
| [*M05.34*](https://www.icd10data.com/ICD10CM/Codes/M00-M99/M05-M14/M05-/M05.34) | Rheumatoid heart disease with rheumatoid arthritis of hand |
| [*M05.341*](https://www.icd10data.com/ICD10CM/Codes/M00-M99/M05-M14/M05-/M05.341) | Rheumatoid heart disease with rheumatoid arthritis of right hand |
| [*M05.342*](https://www.icd10data.com/ICD10CM/Codes/M00-M99/M05-M14/M05-/M05.342) | Rheumatoid heart disease with rheumatoid arthritis of left hand |
| [*M05.349*](https://www.icd10data.com/ICD10CM/Codes/M00-M99/M05-M14/M05-/M05.349) | Rheumatoid heart disease with rheumatoid arthritis of unspecified hand |
| [*M05.35*](https://www.icd10data.com/ICD10CM/Codes/M00-M99/M05-M14/M05-/M05.35) | Rheumatoid heart disease with rheumatoid arthritis of hip |
| [*M05.351*](https://www.icd10data.com/ICD10CM/Codes/M00-M99/M05-M14/M05-/M05.351) | Rheumatoid heart disease with rheumatoid arthritis of right hip |
| [*M05.352*](https://www.icd10data.com/ICD10CM/Codes/M00-M99/M05-M14/M05-/M05.352) | Rheumatoid heart disease with rheumatoid arthritis of left hip |
| [*M05.359*](https://www.icd10data.com/ICD10CM/Codes/M00-M99/M05-M14/M05-/M05.359) | Rheumatoid heart disease with rheumatoid arthritis of unspecified hip |
| [*M05.36*](https://www.icd10data.com/ICD10CM/Codes/M00-M99/M05-M14/M05-/M05.36) | Rheumatoid heart disease with rheumatoid arthritis of knee |
| [*M05.361*](https://www.icd10data.com/ICD10CM/Codes/M00-M99/M05-M14/M05-/M05.361) | Rheumatoid heart disease with rheumatoid arthritis of right knee |
| [*M05.362*](https://www.icd10data.com/ICD10CM/Codes/M00-M99/M05-M14/M05-/M05.362) | Rheumatoid heart disease with rheumatoid arthritis of left knee |
| [*M05.369*](https://www.icd10data.com/ICD10CM/Codes/M00-M99/M05-M14/M05-/M05.369) | Rheumatoid heart disease with rheumatoid arthritis of unspecified knee |
| [*M05.37*](https://www.icd10data.com/ICD10CM/Codes/M00-M99/M05-M14/M05-/M05.37) | Rheumatoid heart disease with rheumatoid arthritis of ankle and foot |
| [*M05.371*](https://www.icd10data.com/ICD10CM/Codes/M00-M99/M05-M14/M05-/M05.371) | Rheumatoid heart disease with rheumatoid arthritis of right ankle and foot |
| [*M05.372*](https://www.icd10data.com/ICD10CM/Codes/M00-M99/M05-M14/M05-/M05.372) | Rheumatoid heart disease with rheumatoid arthritis of left ankle and foot |
| [*M05.379*](https://www.icd10data.com/ICD10CM/Codes/M00-M99/M05-M14/M05-/M05.379) | Rheumatoid heart disease with rheumatoid arthritis of unspecified ankle and foot |
| [*M05.39*](https://www.icd10data.com/ICD10CM/Codes/M00-M99/M05-M14/M05-/M05.39) | …… of multiple sites |
| [*M05.4*](https://www.icd10data.com/ICD10CM/Codes/M00-M99/M05-M14/M05-/M05.4) | Rheumatoid myopathy with rheumatoid arthritis |
| [*M05.40*](https://www.icd10data.com/ICD10CM/Codes/M00-M99/M05-M14/M05-/M05.40) | …… of unspecified site |
| [*M05.41*](https://www.icd10data.com/ICD10CM/Codes/M00-M99/M05-M14/M05-/M05.41) | Rheumatoid myopathy with rheumatoid arthritis of shoulder |
| [*M05.411*](https://www.icd10data.com/ICD10CM/Codes/M00-M99/M05-M14/M05-/M05.411) | Rheumatoid myopathy with rheumatoid arthritis of right shoulder |
| [*M05.412*](https://www.icd10data.com/ICD10CM/Codes/M00-M99/M05-M14/M05-/M05.412) | Rheumatoid myopathy with rheumatoid arthritis of left shoulder |
| [*M05.419*](https://www.icd10data.com/ICD10CM/Codes/M00-M99/M05-M14/M05-/M05.419) | Rheumatoid myopathy with rheumatoid arthritis of unspecified shoulder |
| [*M05.42*](https://www.icd10data.com/ICD10CM/Codes/M00-M99/M05-M14/M05-/M05.42) | Rheumatoid myopathy with rheumatoid arthritis of elbow |
| [*M05.421*](https://www.icd10data.com/ICD10CM/Codes/M00-M99/M05-M14/M05-/M05.421) | Rheumatoid myopathy with rheumatoid arthritis of right elbow |
| [*M05.422*](https://www.icd10data.com/ICD10CM/Codes/M00-M99/M05-M14/M05-/M05.422) | Rheumatoid myopathy with rheumatoid arthritis of left elbow |
| [*M05.429*](https://www.icd10data.com/ICD10CM/Codes/M00-M99/M05-M14/M05-/M05.429) | Rheumatoid myopathy with rheumatoid arthritis of unspecified elbow |
| [*M05.43*](https://www.icd10data.com/ICD10CM/Codes/M00-M99/M05-M14/M05-/M05.43) | Rheumatoid myopathy with rheumatoid arthritis of wrist |
| [*M05.431*](https://www.icd10data.com/ICD10CM/Codes/M00-M99/M05-M14/M05-/M05.431) | Rheumatoid myopathy with rheumatoid arthritis of right wrist |
| [*M05.432*](https://www.icd10data.com/ICD10CM/Codes/M00-M99/M05-M14/M05-/M05.432) | Rheumatoid myopathy with rheumatoid arthritis of left wrist |
| [*M05.439*](https://www.icd10data.com/ICD10CM/Codes/M00-M99/M05-M14/M05-/M05.439) | Rheumatoid myopathy with rheumatoid arthritis of unspecified wrist |
| [*M05.44*](https://www.icd10data.com/ICD10CM/Codes/M00-M99/M05-M14/M05-/M05.44) | Rheumatoid myopathy with rheumatoid arthritis of hand |
| [*M05.441*](https://www.icd10data.com/ICD10CM/Codes/M00-M99/M05-M14/M05-/M05.441) | Rheumatoid myopathy with rheumatoid arthritis of right hand |
| [*M05.442*](https://www.icd10data.com/ICD10CM/Codes/M00-M99/M05-M14/M05-/M05.442) | Rheumatoid myopathy with rheumatoid arthritis of left hand |
| [*M05.449*](https://www.icd10data.com/ICD10CM/Codes/M00-M99/M05-M14/M05-/M05.449) | Rheumatoid myopathy with rheumatoid arthritis of unspecified hand |
| [*M05.45*](https://www.icd10data.com/ICD10CM/Codes/M00-M99/M05-M14/M05-/M05.45) | Rheumatoid myopathy with rheumatoid arthritis of hip |
| [*M05.451*](https://www.icd10data.com/ICD10CM/Codes/M00-M99/M05-M14/M05-/M05.451) | Rheumatoid myopathy with rheumatoid arthritis of right hip |
| [*M05.452*](https://www.icd10data.com/ICD10CM/Codes/M00-M99/M05-M14/M05-/M05.452) | Rheumatoid myopathy with rheumatoid arthritis of left hip |
| [*M05.459*](https://www.icd10data.com/ICD10CM/Codes/M00-M99/M05-M14/M05-/M05.459) | Rheumatoid myopathy with rheumatoid arthritis of unspecified hip |
| [*M05.46*](https://www.icd10data.com/ICD10CM/Codes/M00-M99/M05-M14/M05-/M05.46) | Rheumatoid myopathy with rheumatoid arthritis of knee |
| [*M05.461*](https://www.icd10data.com/ICD10CM/Codes/M00-M99/M05-M14/M05-/M05.461) | Rheumatoid myopathy with rheumatoid arthritis of right knee |
| [*M05.462*](https://www.icd10data.com/ICD10CM/Codes/M00-M99/M05-M14/M05-/M05.462) | Rheumatoid myopathy with rheumatoid arthritis of left knee |
| [*M05.469*](https://www.icd10data.com/ICD10CM/Codes/M00-M99/M05-M14/M05-/M05.469) | Rheumatoid myopathy with rheumatoid arthritis of unspecified knee |
| [*M05.47*](https://www.icd10data.com/ICD10CM/Codes/M00-M99/M05-M14/M05-/M05.47) | Rheumatoid myopathy with rheumatoid arthritis of ankle and foot |
| [*M05.471*](https://www.icd10data.com/ICD10CM/Codes/M00-M99/M05-M14/M05-/M05.471) | Rheumatoid myopathy with rheumatoid arthritis of right ankle and foot |
| [*M05.472*](https://www.icd10data.com/ICD10CM/Codes/M00-M99/M05-M14/M05-/M05.472) | Rheumatoid myopathy with rheumatoid arthritis of left ankle and foot |
| [*M05.479*](https://www.icd10data.com/ICD10CM/Codes/M00-M99/M05-M14/M05-/M05.479) | Rheumatoid myopathy with rheumatoid arthritis of unspecified ankle and foot |
| [*M05.49*](https://www.icd10data.com/ICD10CM/Codes/M00-M99/M05-M14/M05-/M05.49) | …… of multiple sites |
| [*M05.5*](https://www.icd10data.com/ICD10CM/Codes/M00-M99/M05-M14/M05-/M05.5) | Rheumatoid polyneuropathy with rheumatoid arthritis |
| [*M05.50*](https://www.icd10data.com/ICD10CM/Codes/M00-M99/M05-M14/M05-/M05.50) | …… of unspecified site |
| [*M05.51*](https://www.icd10data.com/ICD10CM/Codes/M00-M99/M05-M14/M05-/M05.51) | Rheumatoid polyneuropathy with rheumatoid arthritis of shoulder |
| [*M05.511*](https://www.icd10data.com/ICD10CM/Codes/M00-M99/M05-M14/M05-/M05.511) | Rheumatoid polyneuropathy with rheumatoid arthritis of right shoulder |
| [*M05.512*](https://www.icd10data.com/ICD10CM/Codes/M00-M99/M05-M14/M05-/M05.512) | Rheumatoid polyneuropathy with rheumatoid arthritis of left shoulder |
| [*M05.519*](https://www.icd10data.com/ICD10CM/Codes/M00-M99/M05-M14/M05-/M05.519) | Rheumatoid polyneuropathy with rheumatoid arthritis of unspecified shoulder |
| [*M05.52*](https://www.icd10data.com/ICD10CM/Codes/M00-M99/M05-M14/M05-/M05.52) | Rheumatoid polyneuropathy with rheumatoid arthritis of elbow |
| [*M05.521*](https://www.icd10data.com/ICD10CM/Codes/M00-M99/M05-M14/M05-/M05.521) | Rheumatoid polyneuropathy with rheumatoid arthritis of right elbow |
| [*M05.522*](https://www.icd10data.com/ICD10CM/Codes/M00-M99/M05-M14/M05-/M05.522) | Rheumatoid polyneuropathy with rheumatoid arthritis of left elbow |
| [*M05.529*](https://www.icd10data.com/ICD10CM/Codes/M00-M99/M05-M14/M05-/M05.529) | Rheumatoid polyneuropathy with rheumatoid arthritis of unspecified elbow |
| [*M05.53*](https://www.icd10data.com/ICD10CM/Codes/M00-M99/M05-M14/M05-/M05.53) | Rheumatoid polyneuropathy with rheumatoid arthritis of wrist |
| [*M05.531*](https://www.icd10data.com/ICD10CM/Codes/M00-M99/M05-M14/M05-/M05.531) | Rheumatoid polyneuropathy with rheumatoid arthritis of right wrist |
| [*M05.532*](https://www.icd10data.com/ICD10CM/Codes/M00-M99/M05-M14/M05-/M05.532) | Rheumatoid polyneuropathy with rheumatoid arthritis of left wrist |
| [*M05.539*](https://www.icd10data.com/ICD10CM/Codes/M00-M99/M05-M14/M05-/M05.539) | Rheumatoid polyneuropathy with rheumatoid arthritis of unspecified wrist |
| [*M05.54*](https://www.icd10data.com/ICD10CM/Codes/M00-M99/M05-M14/M05-/M05.54) | Rheumatoid polyneuropathy with rheumatoid arthritis of hand |
| [*M05.541*](https://www.icd10data.com/ICD10CM/Codes/M00-M99/M05-M14/M05-/M05.541) | Rheumatoid polyneuropathy with rheumatoid arthritis of right hand |
| [*M05.542*](https://www.icd10data.com/ICD10CM/Codes/M00-M99/M05-M14/M05-/M05.542) | Rheumatoid polyneuropathy with rheumatoid arthritis of left hand |
| [*M05.549*](https://www.icd10data.com/ICD10CM/Codes/M00-M99/M05-M14/M05-/M05.549) | Rheumatoid polyneuropathy with rheumatoid arthritis of unspecified hand |
| [*M05.55*](https://www.icd10data.com/ICD10CM/Codes/M00-M99/M05-M14/M05-/M05.55) | Rheumatoid polyneuropathy with rheumatoid arthritis of hip |
| [*M05.551*](https://www.icd10data.com/ICD10CM/Codes/M00-M99/M05-M14/M05-/M05.551) | Rheumatoid polyneuropathy with rheumatoid arthritis of right hip |
| [*M05.552*](https://www.icd10data.com/ICD10CM/Codes/M00-M99/M05-M14/M05-/M05.552) | Rheumatoid polyneuropathy with rheumatoid arthritis of left hip |
| [*M05.559*](https://www.icd10data.com/ICD10CM/Codes/M00-M99/M05-M14/M05-/M05.559) | Rheumatoid polyneuropathy with rheumatoid arthritis of unspecified hip |
| [*M05.56*](https://www.icd10data.com/ICD10CM/Codes/M00-M99/M05-M14/M05-/M05.56) | Rheumatoid polyneuropathy with rheumatoid arthritis of knee |
| [*M05.561*](https://www.icd10data.com/ICD10CM/Codes/M00-M99/M05-M14/M05-/M05.561) | Rheumatoid polyneuropathy with rheumatoid arthritis of right knee |
| [*M05.562*](https://www.icd10data.com/ICD10CM/Codes/M00-M99/M05-M14/M05-/M05.562) | Rheumatoid polyneuropathy with rheumatoid arthritis of left knee |
| [*M05.569*](https://www.icd10data.com/ICD10CM/Codes/M00-M99/M05-M14/M05-/M05.569) | Rheumatoid polyneuropathy with rheumatoid arthritis of unspecified knee |
| [*M05.57*](https://www.icd10data.com/ICD10CM/Codes/M00-M99/M05-M14/M05-/M05.57) | Rheumatoid polyneuropathy with rheumatoid arthritis of ankle and foot |
| [*M05.571*](https://www.icd10data.com/ICD10CM/Codes/M00-M99/M05-M14/M05-/M05.571) | Rheumatoid polyneuropathy with rheumatoid arthritis of right ankle and foot |
| [*M05.572*](https://www.icd10data.com/ICD10CM/Codes/M00-M99/M05-M14/M05-/M05.572) | Rheumatoid polyneuropathy with rheumatoid arthritis of left ankle and foot |
| [*M05.579*](https://www.icd10data.com/ICD10CM/Codes/M00-M99/M05-M14/M05-/M05.579) | Rheumatoid polyneuropathy with rheumatoid arthritis of unspecified ankle and foot |
| [*M05.59*](https://www.icd10data.com/ICD10CM/Codes/M00-M99/M05-M14/M05-/M05.59) | …… of multiple sites |
| [*M05.6*](https://www.icd10data.com/ICD10CM/Codes/M00-M99/M05-M14/M05-/M05.6) | Rheumatoid arthritis with involvement of other organs and systems |
| [*M05.60*](https://www.icd10data.com/ICD10CM/Codes/M00-M99/M05-M14/M05-/M05.60) | Rheumatoid arthritis of unspecified site with involvement of other organs and systems |
| [*M05.61*](https://www.icd10data.com/ICD10CM/Codes/M00-M99/M05-M14/M05-/M05.61) | Rheumatoid arthritis of shoulder with involvement of other organs and systems |
| [*M05.611*](https://www.icd10data.com/ICD10CM/Codes/M00-M99/M05-M14/M05-/M05.611) | Rheumatoid arthritis of right shoulder with involvement of other organs and systems |
| [*M05.612*](https://www.icd10data.com/ICD10CM/Codes/M00-M99/M05-M14/M05-/M05.612) | Rheumatoid arthritis of left shoulder with involvement of other organs and systems |
| [*M05.619*](https://www.icd10data.com/ICD10CM/Codes/M00-M99/M05-M14/M05-/M05.619) | Rheumatoid arthritis of unspecified shoulder with involvement of other organs and systems |
| [*M05.62*](https://www.icd10data.com/ICD10CM/Codes/M00-M99/M05-M14/M05-/M05.62) | Rheumatoid arthritis of elbow with involvement of other organs and systems |
| [*M05.621*](https://www.icd10data.com/ICD10CM/Codes/M00-M99/M05-M14/M05-/M05.621) | Rheumatoid arthritis of right elbow with involvement of other organs and systems |
| [*M05.622*](https://www.icd10data.com/ICD10CM/Codes/M00-M99/M05-M14/M05-/M05.622) | Rheumatoid arthritis of left elbow with involvement of other organs and systems |
| [*M05.629*](https://www.icd10data.com/ICD10CM/Codes/M00-M99/M05-M14/M05-/M05.629) | Rheumatoid arthritis of unspecified elbow with involvement of other organs and systems |
| [*M05.63*](https://www.icd10data.com/ICD10CM/Codes/M00-M99/M05-M14/M05-/M05.63) | Rheumatoid arthritis of wrist with involvement of other organs and systems |
| [*M05.631*](https://www.icd10data.com/ICD10CM/Codes/M00-M99/M05-M14/M05-/M05.631) | Rheumatoid arthritis of right wrist with involvement of other organs and systems |
| [*M05.632*](https://www.icd10data.com/ICD10CM/Codes/M00-M99/M05-M14/M05-/M05.632) | Rheumatoid arthritis of left wrist with involvement of other organs and systems |
| [*M05.639*](https://www.icd10data.com/ICD10CM/Codes/M00-M99/M05-M14/M05-/M05.639) | Rheumatoid arthritis of unspecified wrist with involvement of other organs and systems |
| [*M05.64*](https://www.icd10data.com/ICD10CM/Codes/M00-M99/M05-M14/M05-/M05.64) | Rheumatoid arthritis of hand with involvement of other organs and systems |
| [*M05.641*](https://www.icd10data.com/ICD10CM/Codes/M00-M99/M05-M14/M05-/M05.641) | Rheumatoid arthritis of right hand with involvement of other organs and systems |
| [*M05.642*](https://www.icd10data.com/ICD10CM/Codes/M00-M99/M05-M14/M05-/M05.642) | Rheumatoid arthritis of left hand with involvement of other organs and systems |
| [*M05.649*](https://www.icd10data.com/ICD10CM/Codes/M00-M99/M05-M14/M05-/M05.649) | Rheumatoid arthritis of unspecified hand with involvement of other organs and systems |
| [*M05.65*](https://www.icd10data.com/ICD10CM/Codes/M00-M99/M05-M14/M05-/M05.65) | Rheumatoid arthritis of hip with involvement of other organs and systems |
| [*M05.651*](https://www.icd10data.com/ICD10CM/Codes/M00-M99/M05-M14/M05-/M05.651) | Rheumatoid arthritis of right hip with involvement of other organs and systems |
| [*M05.652*](https://www.icd10data.com/ICD10CM/Codes/M00-M99/M05-M14/M05-/M05.652) | Rheumatoid arthritis of left hip with involvement of other organs and systems |
| [*M05.659*](https://www.icd10data.com/ICD10CM/Codes/M00-M99/M05-M14/M05-/M05.659) | Rheumatoid arthritis of unspecified hip with involvement of other organs and systems |
| [*M05.66*](https://www.icd10data.com/ICD10CM/Codes/M00-M99/M05-M14/M05-/M05.66) | Rheumatoid arthritis of knee with involvement of other organs and systems |
| [*M05.661*](https://www.icd10data.com/ICD10CM/Codes/M00-M99/M05-M14/M05-/M05.661) | Rheumatoid arthritis of right knee with involvement of other organs and systems |
| [*M05.662*](https://www.icd10data.com/ICD10CM/Codes/M00-M99/M05-M14/M05-/M05.662) | Rheumatoid arthritis of left knee with involvement of other organs and systems |
| [*M05.669*](https://www.icd10data.com/ICD10CM/Codes/M00-M99/M05-M14/M05-/M05.669) | Rheumatoid arthritis of unspecified knee with involvement of other organs and systems |
| [*M05.67*](https://www.icd10data.com/ICD10CM/Codes/M00-M99/M05-M14/M05-/M05.67) | Rheumatoid arthritis of ankle and foot with involvement of other organs and systems |
| [*M05.671*](https://www.icd10data.com/ICD10CM/Codes/M00-M99/M05-M14/M05-/M05.671) | Rheumatoid arthritis of right ankle and foot with involvement of other organs and systems |
| [*M05.672*](https://www.icd10data.com/ICD10CM/Codes/M00-M99/M05-M14/M05-/M05.672) | Rheumatoid arthritis of left ankle and foot with involvement of other organs and systems |
| [*M05.679*](https://www.icd10data.com/ICD10CM/Codes/M00-M99/M05-M14/M05-/M05.679) | Rheumatoid arthritis of unspecified ankle and foot with involvement of other organs and systems |
| [*M05.69*](https://www.icd10data.com/ICD10CM/Codes/M00-M99/M05-M14/M05-/M05.69) | Rheumatoid arthritis of multiple sites with involvement of other organs and systems |
| [*M05.7*](https://www.icd10data.com/ICD10CM/Codes/M00-M99/M05-M14/M05-/M05.7) | Rheumatoid arthritis with rheumatoid factor without organ or systems involvement |
| [*M05.70*](https://www.icd10data.com/ICD10CM/Codes/M00-M99/M05-M14/M05-/M05.70) | Rheumatoid arthritis with rheumatoid factor of unspecified site without organ or systems involvement |
| [*M05.71*](https://www.icd10data.com/ICD10CM/Codes/M00-M99/M05-M14/M05-/M05.71) | Rheumatoid arthritis with rheumatoid factor of shoulder without organ or systems involvement |
| [*M05.711*](https://www.icd10data.com/ICD10CM/Codes/M00-M99/M05-M14/M05-/M05.711) | Rheumatoid arthritis with rheumatoid factor of right shoulder without organ or systems involvement |
| [*M05.712*](https://www.icd10data.com/ICD10CM/Codes/M00-M99/M05-M14/M05-/M05.712) | Rheumatoid arthritis with rheumatoid factor of left shoulder without organ or systems involvement |
| [*M05.719*](https://www.icd10data.com/ICD10CM/Codes/M00-M99/M05-M14/M05-/M05.719) | Rheumatoid arthritis with rheumatoid factor of unspecified shoulder without organ or systems involvement |
| [*M05.72*](https://www.icd10data.com/ICD10CM/Codes/M00-M99/M05-M14/M05-/M05.72) | Rheumatoid arthritis with rheumatoid factor of elbow without organ or systems involvement |
| [*M05.721*](https://www.icd10data.com/ICD10CM/Codes/M00-M99/M05-M14/M05-/M05.721) | Rheumatoid arthritis with rheumatoid factor of right elbow without organ or systems involvement |
| [*M05.722*](https://www.icd10data.com/ICD10CM/Codes/M00-M99/M05-M14/M05-/M05.722) | Rheumatoid arthritis with rheumatoid factor of left elbow without organ or systems involvement |
| [*M05.729*](https://www.icd10data.com/ICD10CM/Codes/M00-M99/M05-M14/M05-/M05.729) | Rheumatoid arthritis with rheumatoid factor of unspecified elbow without organ or systems involvement |
| [*M05.73*](https://www.icd10data.com/ICD10CM/Codes/M00-M99/M05-M14/M05-/M05.73) | Rheumatoid arthritis with rheumatoid factor of wrist without organ or systems involvement |
| [*M05.731*](https://www.icd10data.com/ICD10CM/Codes/M00-M99/M05-M14/M05-/M05.731) | Rheumatoid arthritis with rheumatoid factor of right wrist without organ or systems involvement |
| [*M05.732*](https://www.icd10data.com/ICD10CM/Codes/M00-M99/M05-M14/M05-/M05.732) | Rheumatoid arthritis with rheumatoid factor of left wrist without organ or systems involvement |
| [*M05.739*](https://www.icd10data.com/ICD10CM/Codes/M00-M99/M05-M14/M05-/M05.739) | Rheumatoid arthritis with rheumatoid factor of unspecified wrist without organ or systems involvement |
| [*M05.74*](https://www.icd10data.com/ICD10CM/Codes/M00-M99/M05-M14/M05-/M05.74) | Rheumatoid arthritis with rheumatoid factor of hand without organ or systems involvement |
| [*M05.741*](https://www.icd10data.com/ICD10CM/Codes/M00-M99/M05-M14/M05-/M05.741) | Rheumatoid arthritis with rheumatoid factor of right hand without organ or systems involvement |
| [*M05.742*](https://www.icd10data.com/ICD10CM/Codes/M00-M99/M05-M14/M05-/M05.742) | Rheumatoid arthritis with rheumatoid factor of left hand without organ or systems involvement |
| [*M05.749*](https://www.icd10data.com/ICD10CM/Codes/M00-M99/M05-M14/M05-/M05.749) | Rheumatoid arthritis with rheumatoid factor of unspecified hand without organ or systems involvement |
| [*M05.75*](https://www.icd10data.com/ICD10CM/Codes/M00-M99/M05-M14/M05-/M05.75) | Rheumatoid arthritis with rheumatoid factor of hip without organ or systems involvement |
| [*M05.751*](https://www.icd10data.com/ICD10CM/Codes/M00-M99/M05-M14/M05-/M05.751) | Rheumatoid arthritis with rheumatoid factor of right hip without organ or systems involvement |
| [*M05.752*](https://www.icd10data.com/ICD10CM/Codes/M00-M99/M05-M14/M05-/M05.752) | Rheumatoid arthritis with rheumatoid factor of left hip without organ or systems involvement |
| [*M05.759*](https://www.icd10data.com/ICD10CM/Codes/M00-M99/M05-M14/M05-/M05.759) | Rheumatoid arthritis with rheumatoid factor of unspecified hip without organ or systems involvement |
| [*M05.76*](https://www.icd10data.com/ICD10CM/Codes/M00-M99/M05-M14/M05-/M05.76) | Rheumatoid arthritis with rheumatoid factor of knee without organ or systems involvement |
| [*M05.761*](https://www.icd10data.com/ICD10CM/Codes/M00-M99/M05-M14/M05-/M05.761) | Rheumatoid arthritis with rheumatoid factor of right knee without organ or systems involvement |
| [*M05.762*](https://www.icd10data.com/ICD10CM/Codes/M00-M99/M05-M14/M05-/M05.762) | Rheumatoid arthritis with rheumatoid factor of left knee without organ or systems involvement |
| [*M05.769*](https://www.icd10data.com/ICD10CM/Codes/M00-M99/M05-M14/M05-/M05.769) | Rheumatoid arthritis with rheumatoid factor of unspecified knee without organ or systems involvement |
| [*M05.77*](https://www.icd10data.com/ICD10CM/Codes/M00-M99/M05-M14/M05-/M05.77) | Rheumatoid arthritis with rheumatoid factor of ankle and foot without organ or systems involvement |
| [*M05.771*](https://www.icd10data.com/ICD10CM/Codes/M00-M99/M05-M14/M05-/M05.771) | Rheumatoid arthritis with rheumatoid factor of right ankle and foot without organ or systems involvement |
| [*M05.772*](https://www.icd10data.com/ICD10CM/Codes/M00-M99/M05-M14/M05-/M05.772) | Rheumatoid arthritis with rheumatoid factor of left ankle and foot without organ or systems involvement |
| [*M05.779*](https://www.icd10data.com/ICD10CM/Codes/M00-M99/M05-M14/M05-/M05.779) | Rheumatoid arthritis with rheumatoid factor of unspecified ankle and foot without organ or systems involvement |
| [*M05.79*](https://www.icd10data.com/ICD10CM/Codes/M00-M99/M05-M14/M05-/M05.79) | Rheumatoid arthritis with rheumatoid factor of multiple sites without organ or systems involvement |
| [*M05.7A*](https://www.icd10data.com/ICD10CM/Codes/M00-M99/M05-M14/M05-/M05.7A) | Rheumatoid arthritis with rheumatoid factor of other specified site without organ or systems involvement |
| [*M05.8*](https://www.icd10data.com/ICD10CM/Codes/M00-M99/M05-M14/M05-/M05.8) | Other rheumatoid arthritis with rheumatoid factor |
| [*M05.80*](https://www.icd10data.com/ICD10CM/Codes/M00-M99/M05-M14/M05-/M05.80) | …… of unspecified site |
| [*M05.81*](https://www.icd10data.com/ICD10CM/Codes/M00-M99/M05-M14/M05-/M05.81) | Other rheumatoid arthritis with rheumatoid factor of shoulder |
| [*M05.811*](https://www.icd10data.com/ICD10CM/Codes/M00-M99/M05-M14/M05-/M05.811) | Other rheumatoid arthritis with rheumatoid factor of right shoulder |
| [*M05.812*](https://www.icd10data.com/ICD10CM/Codes/M00-M99/M05-M14/M05-/M05.812) | Other rheumatoid arthritis with rheumatoid factor of left shoulder |
| [*M05.819*](https://www.icd10data.com/ICD10CM/Codes/M00-M99/M05-M14/M05-/M05.819) | Other rheumatoid arthritis with rheumatoid factor of unspecified shoulder |
| [*M05.82*](https://www.icd10data.com/ICD10CM/Codes/M00-M99/M05-M14/M05-/M05.82) | Other rheumatoid arthritis with rheumatoid factor of elbow |
| [*M05.821*](https://www.icd10data.com/ICD10CM/Codes/M00-M99/M05-M14/M05-/M05.821) | Other rheumatoid arthritis with rheumatoid factor of right elbow |
| [*M05.822*](https://www.icd10data.com/ICD10CM/Codes/M00-M99/M05-M14/M05-/M05.822) | Other rheumatoid arthritis with rheumatoid factor of left elbow |
| [*M05.829*](https://www.icd10data.com/ICD10CM/Codes/M00-M99/M05-M14/M05-/M05.829) | Other rheumatoid arthritis with rheumatoid factor of unspecified elbow |
| [*M05.83*](https://www.icd10data.com/ICD10CM/Codes/M00-M99/M05-M14/M05-/M05.83) | Other rheumatoid arthritis with rheumatoid factor of wrist |
| [*M05.831*](https://www.icd10data.com/ICD10CM/Codes/M00-M99/M05-M14/M05-/M05.831) | Other rheumatoid arthritis with rheumatoid factor of right wrist |
| [*M05.832*](https://www.icd10data.com/ICD10CM/Codes/M00-M99/M05-M14/M05-/M05.832) | Other rheumatoid arthritis with rheumatoid factor of left wrist |
| [*M05.839*](https://www.icd10data.com/ICD10CM/Codes/M00-M99/M05-M14/M05-/M05.839) | Other rheumatoid arthritis with rheumatoid factor of unspecified wrist |
| [*M05.84*](https://www.icd10data.com/ICD10CM/Codes/M00-M99/M05-M14/M05-/M05.84) | Other rheumatoid arthritis with rheumatoid factor of hand |
| [*M05.841*](https://www.icd10data.com/ICD10CM/Codes/M00-M99/M05-M14/M05-/M05.841) | Other rheumatoid arthritis with rheumatoid factor of right hand |
| [*M05.842*](https://www.icd10data.com/ICD10CM/Codes/M00-M99/M05-M14/M05-/M05.842) | Other rheumatoid arthritis with rheumatoid factor of left hand |
| [*M05.849*](https://www.icd10data.com/ICD10CM/Codes/M00-M99/M05-M14/M05-/M05.849) | Other rheumatoid arthritis with rheumatoid factor of unspecified hand |
| [*M05.85*](https://www.icd10data.com/ICD10CM/Codes/M00-M99/M05-M14/M05-/M05.85) | Other rheumatoid arthritis with rheumatoid factor of hip |
| [*M05.851*](https://www.icd10data.com/ICD10CM/Codes/M00-M99/M05-M14/M05-/M05.851) | Other rheumatoid arthritis with rheumatoid factor of right hip |
| [*M05.852*](https://www.icd10data.com/ICD10CM/Codes/M00-M99/M05-M14/M05-/M05.852) | Other rheumatoid arthritis with rheumatoid factor of left hip |
| [*M05.859*](https://www.icd10data.com/ICD10CM/Codes/M00-M99/M05-M14/M05-/M05.859) | Other rheumatoid arthritis with rheumatoid factor of unspecified hip |
| [*M05.86*](https://www.icd10data.com/ICD10CM/Codes/M00-M99/M05-M14/M05-/M05.86) | Other rheumatoid arthritis with rheumatoid factor of knee |
| [*M05.861*](https://www.icd10data.com/ICD10CM/Codes/M00-M99/M05-M14/M05-/M05.861) | Other rheumatoid arthritis with rheumatoid factor of right knee |
| [*M05.862*](https://www.icd10data.com/ICD10CM/Codes/M00-M99/M05-M14/M05-/M05.862) | Other rheumatoid arthritis with rheumatoid factor of left knee |
| [*M05.869*](https://www.icd10data.com/ICD10CM/Codes/M00-M99/M05-M14/M05-/M05.869) | Other rheumatoid arthritis with rheumatoid factor of unspecified knee |
| [*M05.87*](https://www.icd10data.com/ICD10CM/Codes/M00-M99/M05-M14/M05-/M05.87) | Other rheumatoid arthritis with rheumatoid factor of ankle and foot |
| [*M05.871*](https://www.icd10data.com/ICD10CM/Codes/M00-M99/M05-M14/M05-/M05.871) | Other rheumatoid arthritis with rheumatoid factor of right ankle and foot |
| [*M05.872*](https://www.icd10data.com/ICD10CM/Codes/M00-M99/M05-M14/M05-/M05.872) | Other rheumatoid arthritis with rheumatoid factor of left ankle and foot |
| [*M05.879*](https://www.icd10data.com/ICD10CM/Codes/M00-M99/M05-M14/M05-/M05.879) | Other rheumatoid arthritis with rheumatoid factor of unspecified ankle and foot |
| [*M05.89*](https://www.icd10data.com/ICD10CM/Codes/M00-M99/M05-M14/M05-/M05.89) | …… of multiple sites |
| [*M05.8A*](https://www.icd10data.com/ICD10CM/Codes/M00-M99/M05-M14/M05-/M05.8A) | …… of other specified site |
| [*M05.9*](https://www.icd10data.com/ICD10CM/Codes/M00-M99/M05-M14/M05-/M05.9) | Rheumatoid arthritis with rheumatoid factor, unspecified |

**1b – ICD-10 codes for Juvenile arthritis**

| [*M08*](https://www.icd10data.com/ICD10CM/Codes/M00-M99/M05-M14/M08-/M08) | **Juvenile arthritis** |
| --- | --- |
| [*M08.0*](https://www.icd10data.com/ICD10CM/Codes/M00-M99/M05-M14/M08-/M08.0) | Unspecified juvenile rheumatoid arthritis |
| [*M*](https://www.icd10data.com/ICD10CM/Codes/M00-M99/M05-M14/M08-/M08.00)*08.00* | …… of unspecified site |
| [*M08.01*](https://www.icd10data.com/ICD10CM/Codes/M00-M99/M05-M14/M08-/M08.01) | Unspecified juvenile rheumatoid arthritis, shoulder |
| [*M08.011*](https://www.icd10data.com/ICD10CM/Codes/M00-M99/M05-M14/M08-/M08.011) | Unspecified juvenile rheumatoid arthritis, right shoulder |
| [*M08.012*](https://www.icd10data.com/ICD10CM/Codes/M00-M99/M05-M14/M08-/M08.012) | Unspecified juvenile rheumatoid arthritis, left shoulder |
| [*M08.019*](https://www.icd10data.com/ICD10CM/Codes/M00-M99/M05-M14/M08-/M08.019) | Unspecified juvenile rheumatoid arthritis, unspecified shoulder |
| [*M08.02*](https://www.icd10data.com/ICD10CM/Codes/M00-M99/M05-M14/M08-/M08.02) | Unspecified juvenile rheumatoid arthritis of elbow |
| [*M0*](https://www.icd10data.com/ICD10CM/Codes/M00-M99/M05-M14/M08-/M08.021)*8.021* | Unspecified juvenile rheumatoid arthritis, right elbow |
| [*M08.022*](https://www.icd10data.com/ICD10CM/Codes/M00-M99/M05-M14/M08-/M08.022) | Unspecified juvenile rheumatoid arthritis, left elbow |
| [*M08.029*](https://www.icd10data.com/ICD10CM/Codes/M00-M99/M05-M14/M08-/M08.029) | Unspecified juvenile rheumatoid arthritis, unspecified elbow |
| [*M08.03*](https://www.icd10data.com/ICD10CM/Codes/M00-M99/M05-M14/M08-/M08.03) | Unspecified juvenile rheumatoid arthritis, wrist |
| [*M08.031*](https://www.icd10data.com/ICD10CM/Codes/M00-M99/M05-M14/M08-/M08.031) | Unspecified juvenile rheumatoid arthritis, right wrist |
| [*M08.032*](https://www.icd10data.com/ICD10CM/Codes/M00-M99/M05-M14/M08-/M08.032) | Unspecified juvenile rheumatoid arthritis, left wrist |
| [*M08.039*](https://www.icd10data.com/ICD10CM/Codes/M00-M99/M05-M14/M08-/M08.039) | Unspecified juvenile rheumatoid arthritis, unspecified wrist |
| [*M08.04*](https://www.icd10data.com/ICD10CM/Codes/M00-M99/M05-M14/M08-/M08.04) | Unspecified juvenile rheumatoid arthritis, hand |
| [*M08.041*](https://www.icd10data.com/ICD10CM/Codes/M00-M99/M05-M14/M08-/M08.041) | Unspecified juvenile rheumatoid arthritis, right hand |
| [*M08.042*](https://www.icd10data.com/ICD10CM/Codes/M00-M99/M05-M14/M08-/M08.042) | Unspecified juvenile rheumatoid arthritis, left hand |
| [*M08.049*](https://www.icd10data.com/ICD10CM/Codes/M00-M99/M05-M14/M08-/M08.049) | Unspecified juvenile rheumatoid arthritis, unspecified hand |
| [*M08.05*](https://www.icd10data.com/ICD10CM/Codes/M00-M99/M05-M14/M08-/M08.05) | Unspecified juvenile rheumatoid arthritis, hip |
| [*M08.051*](https://www.icd10data.com/ICD10CM/Codes/M00-M99/M05-M14/M08-/M08.051) | Unspecified juvenile rheumatoid arthritis, right hip |
| [*M08.052*](https://www.icd10data.com/ICD10CM/Codes/M00-M99/M05-M14/M08-/M08.052) | Unspecified juvenile rheumatoid arthritis, left hip |
| [*M08.059*](https://www.icd10data.com/ICD10CM/Codes/M00-M99/M05-M14/M08-/M08.059) | Unspecified juvenile rheumatoid arthritis, unspecified hip |
| [*M08.06*](https://www.icd10data.com/ICD10CM/Codes/M00-M99/M05-M14/M08-/M08.06) | Unspecified juvenile rheumatoid arthritis, knee |
| [*M08.061*](https://www.icd10data.com/ICD10CM/Codes/M00-M99/M05-M14/M08-/M08.061) | Unspecified juvenile rheumatoid arthritis, right knee |
| [*M08.062*](https://www.icd10data.com/ICD10CM/Codes/M00-M99/M05-M14/M08-/M08.062) | Unspecified juvenile rheumatoid arthritis, left knee |
| [*M08.069*](https://www.icd10data.com/ICD10CM/Codes/M00-M99/M05-M14/M08-/M08.069) | Unspecified juvenile rheumatoid arthritis, unspecified knee |
| [*M08.07*](https://www.icd10data.com/ICD10CM/Codes/M00-M99/M05-M14/M08-/M08.07) | Unspecified juvenile rheumatoid arthritis, ankle and foot |
| [*M08.071*](https://www.icd10data.com/ICD10CM/Codes/M00-M99/M05-M14/M08-/M08.071) | Unspecified juvenile rheumatoid arthritis, right ankle and foot |
| [*M08.072*](https://www.icd10data.com/ICD10CM/Codes/M00-M99/M05-M14/M08-/M08.072) | Unspecified juvenile rheumatoid arthritis, left ankle and foot |
| [*M08.079*](https://www.icd10data.com/ICD10CM/Codes/M00-M99/M05-M14/M08-/M08.079) | Unspecified juvenile rheumatoid arthritis, unspecified ankle and foot |
| [*M08.08*](https://www.icd10data.com/ICD10CM/Codes/M00-M99/M05-M14/M08-/M08.08) | …… vertebrae |
| [*M08.09*](https://www.icd10data.com/ICD10CM/Codes/M00-M99/M05-M14/M08-/M08.09) | …… multiple sites |
| [*M08.0A*](https://www.icd10data.com/ICD10CM/Codes/M00-M99/M05-M14/M08-/M08.0A) | …… other specified site |
| [*M08.1*](https://www.icd10data.com/ICD10CM/Codes/M00-M99/M05-M14/M08-/M08.1) | Juvenile ankylosing spondylitis |
| [*M08.2*](https://www.icd10data.com/ICD10CM/Codes/M00-M99/M05-M14/M08-/M08.2) | Juvenile rheumatoid arthritis with systemic onset |
| [*M08.20*](https://www.icd10data.com/ICD10CM/Codes/M00-M99/M05-M14/M08-/M08.20) | …… unspecified site |
| [*M08.21*](https://www.icd10data.com/ICD10CM/Codes/M00-M99/M05-M14/M08-/M08.21) | Juvenile rheumatoid arthritis with systemic onset, shoulder |
| [*M08.211*](https://www.icd10data.com/ICD10CM/Codes/M00-M99/M05-M14/M08-/M08.211) | Juvenile rheumatoid arthritis with systemic onset, right shoulder |
| [*M08.212*](https://www.icd10data.com/ICD10CM/Codes/M00-M99/M05-M14/M08-/M08.212) | Juvenile rheumatoid arthritis with systemic onset, left shoulder |
| [*M08.219*](https://www.icd10data.com/ICD10CM/Codes/M00-M99/M05-M14/M08-/M08.219) | Juvenile rheumatoid arthritis with systemic onset, unspecified shoulder |
| [*M08.22*](https://www.icd10data.com/ICD10CM/Codes/M00-M99/M05-M14/M08-/M08.22) | Juvenile rheumatoid arthritis with systemic onset, elbow |
| [*M08.221*](https://www.icd10data.com/ICD10CM/Codes/M00-M99/M05-M14/M08-/M08.221) | Juvenile rheumatoid arthritis with systemic onset, right elbow |
| [*M08.222*](https://www.icd10data.com/ICD10CM/Codes/M00-M99/M05-M14/M08-/M08.222) | Juvenile rheumatoid arthritis with systemic onset, left elbow |
| [*M08.229*](https://www.icd10data.com/ICD10CM/Codes/M00-M99/M05-M14/M08-/M08.229) | Juvenile rheumatoid arthritis with systemic onset, unspecified elbow |
| [*M08.23*](https://www.icd10data.com/ICD10CM/Codes/M00-M99/M05-M14/M08-/M08.23) | Juvenile rheumatoid arthritis with systemic onset, wrist |
| [*M08.231*](https://www.icd10data.com/ICD10CM/Codes/M00-M99/M05-M14/M08-/M08.231) | Juvenile rheumatoid arthritis with systemic onset, right wrist |
| [*M08.232*](https://www.icd10data.com/ICD10CM/Codes/M00-M99/M05-M14/M08-/M08.232) | Juvenile rheumatoid arthritis with systemic onset, left wrist |
| [*M08.239*](https://www.icd10data.com/ICD10CM/Codes/M00-M99/M05-M14/M08-/M08.239) | Juvenile rheumatoid arthritis with systemic onset, unspecified wrist |
| [*M08.24*](https://www.icd10data.com/ICD10CM/Codes/M00-M99/M05-M14/M08-/M08.24) | Juvenile rheumatoid arthritis with systemic onset, hand |
| [*M08.241*](https://www.icd10data.com/ICD10CM/Codes/M00-M99/M05-M14/M08-/M08.241) | Juvenile rheumatoid arthritis with systemic onset, right hand |
| [*M08.242*](https://www.icd10data.com/ICD10CM/Codes/M00-M99/M05-M14/M08-/M08.242) | Juvenile rheumatoid arthritis with systemic onset, left hand |
| [*M08.249*](https://www.icd10data.com/ICD10CM/Codes/M00-M99/M05-M14/M08-/M08.249) | Juvenile rheumatoid arthritis with systemic onset, unspecified hand |
| [*M08.25*](https://www.icd10data.com/ICD10CM/Codes/M00-M99/M05-M14/M08-/M08.25) | Juvenile rheumatoid arthritis with systemic onset, hip |
| [*M08.251*](https://www.icd10data.com/ICD10CM/Codes/M00-M99/M05-M14/M08-/M08.251) | Juvenile rheumatoid arthritis with systemic onset, right hip |
| [*M08.252*](https://www.icd10data.com/ICD10CM/Codes/M00-M99/M05-M14/M08-/M08.252) | Juvenile rheumatoid arthritis with systemic onset, left hip |
| [*M08.259*](https://www.icd10data.com/ICD10CM/Codes/M00-M99/M05-M14/M08-/M08.259) | Juvenile rheumatoid arthritis with systemic onset, unspecified hip |
| [*M08.26*](https://www.icd10data.com/ICD10CM/Codes/M00-M99/M05-M14/M08-/M08.26) | Juvenile rheumatoid arthritis with systemic onset, knee |
| [*M08.261*](https://www.icd10data.com/ICD10CM/Codes/M00-M99/M05-M14/M08-/M08.261) | Juvenile rheumatoid arthritis with systemic onset, right knee |
| [*M08.262*](https://www.icd10data.com/ICD10CM/Codes/M00-M99/M05-M14/M08-/M08.262) | Juvenile rheumatoid arthritis with systemic onset, left knee |
| [*M08.269*](https://www.icd10data.com/ICD10CM/Codes/M00-M99/M05-M14/M08-/M08.269) | Juvenile rheumatoid arthritis with systemic onset, unspecified knee |
| [*M08.27*](https://www.icd10data.com/ICD10CM/Codes/M00-M99/M05-M14/M08-/M08.27) | Juvenile rheumatoid arthritis with systemic onset, ankle and foot |
| [*M08.271*](https://www.icd10data.com/ICD10CM/Codes/M00-M99/M05-M14/M08-/M08.271) | Juvenile rheumatoid arthritis with systemic onset, right ankle and foot |
| [*M08.272*](https://www.icd10data.com/ICD10CM/Codes/M00-M99/M05-M14/M08-/M08.272) | Juvenile rheumatoid arthritis with systemic onset, left ankle and foot |
| [*M08.279*](https://www.icd10data.com/ICD10CM/Codes/M00-M99/M05-M14/M08-/M08.279) | Juvenile rheumatoid arthritis with systemic onset, unspecified ankle and foot |
| [*M08.28*](https://www.icd10data.com/ICD10CM/Codes/M00-M99/M05-M14/M08-/M08.28) | …… vertebrae |
| [*M08.29*](https://www.icd10data.com/ICD10CM/Codes/M00-M99/M05-M14/M08-/M08.29) | …… multiple sites |
| [*M08.2A*](https://www.icd10data.com/ICD10CM/Codes/M00-M99/M05-M14/M08-/M08.2A) | …… other specified site |
| [*M08.3*](https://www.icd10data.com/ICD10CM/Codes/M00-M99/M05-M14/M08-/M08.3) | Juvenile rheumatoid polyarthritis (seronegative) |
| [*M08.4*](https://www.icd10data.com/ICD10CM/Codes/M00-M99/M05-M14/M08-/M08.4) | Pauciarticular juvenile rheumatoid arthritis |
| [*M08.40*](https://www.icd10data.com/ICD10CM/Codes/M00-M99/M05-M14/M08-/M08.40) | …… unspecified site |
| [*M08.41*](https://www.icd10data.com/ICD10CM/Codes/M00-M99/M05-M14/M08-/M08.41) | Pauciarticular juvenile rheumatoid arthritis, shoulder |
| [*M08.411*](https://www.icd10data.com/ICD10CM/Codes/M00-M99/M05-M14/M08-/M08.411) | Pauciarticular juvenile rheumatoid arthritis, right shoulder |
| [*M08.412*](https://www.icd10data.com/ICD10CM/Codes/M00-M99/M05-M14/M08-/M08.412) | Pauciarticular juvenile rheumatoid arthritis, left shoulder |
| [*M08.419*](https://www.icd10data.com/ICD10CM/Codes/M00-M99/M05-M14/M08-/M08.419) | Pauciarticular juvenile rheumatoid arthritis, unspecified shoulder |
| [*M08.42*](https://www.icd10data.com/ICD10CM/Codes/M00-M99/M05-M14/M08-/M08.42) | Pauciarticular juvenile rheumatoid arthritis, elbow |
| [*M08.421*](https://www.icd10data.com/ICD10CM/Codes/M00-M99/M05-M14/M08-/M08.421) | Pauciarticular juvenile rheumatoid arthritis, right elbow |
| [*M08.422*](https://www.icd10data.com/ICD10CM/Codes/M00-M99/M05-M14/M08-/M08.422) | Pauciarticular juvenile rheumatoid arthritis, left elbow |
| [*M08.429*](https://www.icd10data.com/ICD10CM/Codes/M00-M99/M05-M14/M08-/M08.429) | Pauciarticular juvenile rheumatoid arthritis, unspecified elbow |
| [*M08.43*](https://www.icd10data.com/ICD10CM/Codes/M00-M99/M05-M14/M08-/M08.43) | Pauciarticular juvenile rheumatoid arthritis, wrist |
| [*M08.431*](https://www.icd10data.com/ICD10CM/Codes/M00-M99/M05-M14/M08-/M08.431) | Pauciarticular juvenile rheumatoid arthritis, right wrist |
| [*M08.432*](https://www.icd10data.com/ICD10CM/Codes/M00-M99/M05-M14/M08-/M08.432) | Pauciarticular juvenile rheumatoid arthritis, left wrist |
| [*M08.439*](https://www.icd10data.com/ICD10CM/Codes/M00-M99/M05-M14/M08-/M08.439) | Pauciarticular juvenile rheumatoid arthritis, unspecified wrist |
| [*M08.44*](https://www.icd10data.com/ICD10CM/Codes/M00-M99/M05-M14/M08-/M08.44) | Pauciarticular juvenile rheumatoid arthritis, hand |
| [*M08.441*](https://www.icd10data.com/ICD10CM/Codes/M00-M99/M05-M14/M08-/M08.441) | Pauciarticular juvenile rheumatoid arthritis, right hand |
| [*M08.442*](https://www.icd10data.com/ICD10CM/Codes/M00-M99/M05-M14/M08-/M08.442) | Pauciarticular juvenile rheumatoid arthritis, left hand |
| [*M08.449*](https://www.icd10data.com/ICD10CM/Codes/M00-M99/M05-M14/M08-/M08.449) | Pauciarticular juvenile rheumatoid arthritis, unspecified hand |
| [*M08.45*](https://www.icd10data.com/ICD10CM/Codes/M00-M99/M05-M14/M08-/M08.45) | Pauciarticular juvenile rheumatoid arthritis, hip |
| [*M08.451*](https://www.icd10data.com/ICD10CM/Codes/M00-M99/M05-M14/M08-/M08.451) | Pauciarticular juvenile rheumatoid arthritis, right hip |
| [*M08.452*](https://www.icd10data.com/ICD10CM/Codes/M00-M99/M05-M14/M08-/M08.452) | Pauciarticular juvenile rheumatoid arthritis, left hip |
| [*M08.459*](https://www.icd10data.com/ICD10CM/Codes/M00-M99/M05-M14/M08-/M08.459) | Pauciarticular juvenile rheumatoid arthritis, unspecified hip |
| [*M08.46*](https://www.icd10data.com/ICD10CM/Codes/M00-M99/M05-M14/M08-/M08.46) | Pauciarticular juvenile rheumatoid arthritis, knee |
| [*M08.461*](https://www.icd10data.com/ICD10CM/Codes/M00-M99/M05-M14/M08-/M08.461) | Pauciarticular juvenile rheumatoid arthritis, right knee |
| [*M08.462*](https://www.icd10data.com/ICD10CM/Codes/M00-M99/M05-M14/M08-/M08.462) | Pauciarticular juvenile rheumatoid arthritis, left knee |
| [*M08.469*](https://www.icd10data.com/ICD10CM/Codes/M00-M99/M05-M14/M08-/M08.469) | Pauciarticular juvenile rheumatoid arthritis, unspecified knee |
| [*M08.47*](https://www.icd10data.com/ICD10CM/Codes/M00-M99/M05-M14/M08-/M08.47) | Pauciarticular juvenile rheumatoid arthritis, ankle and foot |
| [*M08.471*](https://www.icd10data.com/ICD10CM/Codes/M00-M99/M05-M14/M08-/M08.471) | Pauciarticular juvenile rheumatoid arthritis, right ankle and foot |
| [*M08.472*](https://www.icd10data.com/ICD10CM/Codes/M00-M99/M05-M14/M08-/M08.472) | Pauciarticular juvenile rheumatoid arthritis, left ankle and foot |
| [*M08.479*](https://www.icd10data.com/ICD10CM/Codes/M00-M99/M05-M14/M08-/M08.479) | Pauciarticular juvenile rheumatoid arthritis, unspecified ankle and foot |
| [*M08.48*](https://www.icd10data.com/ICD10CM/Codes/M00-M99/M05-M14/M08-/M08.48) | …… vertebrae |
| [*M08.4A*](https://www.icd10data.com/ICD10CM/Codes/M00-M99/M05-M14/M08-/M08.4A) | …… other specified site |
| [*M08.8*](https://www.icd10data.com/ICD10CM/Codes/M00-M99/M05-M14/M08-/M08.8) | Other juvenile arthritis |
| [*M08.80*](https://www.icd10data.com/ICD10CM/Codes/M00-M99/M05-M14/M08-/M08.80) | …… unspecified site |
| [*M08.81*](https://www.icd10data.com/ICD10CM/Codes/M00-M99/M05-M14/M08-/M08.81) | Other juvenile arthritis, shoulder |
| [*M08.811*](https://www.icd10data.com/ICD10CM/Codes/M00-M99/M05-M14/M08-/M08.811) | Other juvenile arthritis, right shoulder |
| [*M08.812*](https://www.icd10data.com/ICD10CM/Codes/M00-M99/M05-M14/M08-/M08.812) | Other juvenile arthritis, left shoulder |
| [*M08.819*](https://www.icd10data.com/ICD10CM/Codes/M00-M99/M05-M14/M08-/M08.819) | Other juvenile arthritis, unspecified shoulder |
| [*M08.82*](https://www.icd10data.com/ICD10CM/Codes/M00-M99/M05-M14/M08-/M08.82) | Other juvenile arthritis, elbow |
| [*M08.821*](https://www.icd10data.com/ICD10CM/Codes/M00-M99/M05-M14/M08-/M08.821) | Other juvenile arthritis, right elbow |
| [*M08.822*](https://www.icd10data.com/ICD10CM/Codes/M00-M99/M05-M14/M08-/M08.822) | Other juvenile arthritis, left elbow |
| [*M08.829*](https://www.icd10data.com/ICD10CM/Codes/M00-M99/M05-M14/M08-/M08.829) | Other juvenile arthritis, unspecified elbow |
| [*M08.83*](https://www.icd10data.com/ICD10CM/Codes/M00-M99/M05-M14/M08-/M08.83) | Other juvenile arthritis, wrist |
| [*M08.831*](https://www.icd10data.com/ICD10CM/Codes/M00-M99/M05-M14/M08-/M08.831) | Other juvenile arthritis, right wrist |
| [*M08.832*](https://www.icd10data.com/ICD10CM/Codes/M00-M99/M05-M14/M08-/M08.832) | Other juvenile arthritis, left wrist |
| [*M08.839*](https://www.icd10data.com/ICD10CM/Codes/M00-M99/M05-M14/M08-/M08.839) | Other juvenile arthritis, unspecified wrist |
| [*M08.84*](https://www.icd10data.com/ICD10CM/Codes/M00-M99/M05-M14/M08-/M08.84) | Other juvenile arthritis, hand |
| [*M08.841*](https://www.icd10data.com/ICD10CM/Codes/M00-M99/M05-M14/M08-/M08.841) | Other juvenile arthritis, right hand |
| [*M08.842*](https://www.icd10data.com/ICD10CM/Codes/M00-M99/M05-M14/M08-/M08.842) | Other juvenile arthritis, left hand |
| [*M08.849*](https://www.icd10data.com/ICD10CM/Codes/M00-M99/M05-M14/M08-/M08.849) | Other juvenile arthritis, unspecified hand |
| [*M08.85*](https://www.icd10data.com/ICD10CM/Codes/M00-M99/M05-M14/M08-/M08.85) | Other juvenile arthritis, hip |
| [*M08.851*](https://www.icd10data.com/ICD10CM/Codes/M00-M99/M05-M14/M08-/M08.851) | Other juvenile arthritis, right hip |
| [*M08.852*](https://www.icd10data.com/ICD10CM/Codes/M00-M99/M05-M14/M08-/M08.852) | Other juvenile arthritis, left hip |
| [*M08.859*](https://www.icd10data.com/ICD10CM/Codes/M00-M99/M05-M14/M08-/M08.859) | Other juvenile arthritis, unspecified hip |
| [*M08.86*](https://www.icd10data.com/ICD10CM/Codes/M00-M99/M05-M14/M08-/M08.86) | Other juvenile arthritis, knee |
| [*M08.861*](https://www.icd10data.com/ICD10CM/Codes/M00-M99/M05-M14/M08-/M08.861) | Other juvenile arthritis, right knee |
| [*M08.862*](https://www.icd10data.com/ICD10CM/Codes/M00-M99/M05-M14/M08-/M08.862) | Other juvenile arthritis, left knee |
| [*M08.869*](https://www.icd10data.com/ICD10CM/Codes/M00-M99/M05-M14/M08-/M08.869) | Other juvenile arthritis, unspecified knee |
| [*M08.87*](https://www.icd10data.com/ICD10CM/Codes/M00-M99/M05-M14/M08-/M08.87) | Other juvenile arthritis, ankle and foot |
| [*M08.871*](https://www.icd10data.com/ICD10CM/Codes/M00-M99/M05-M14/M08-/M08.871) | Other juvenile arthritis, right ankle and foot |
| [*M08.872*](https://www.icd10data.com/ICD10CM/Codes/M00-M99/M05-M14/M08-/M08.872) | Other juvenile arthritis, left ankle and foot |
| [*M08.879*](https://www.icd10data.com/ICD10CM/Codes/M00-M99/M05-M14/M08-/M08.879) | Other juvenile arthritis, unspecified ankle and foot |
| [*M08.88*](https://www.icd10data.com/ICD10CM/Codes/M00-M99/M05-M14/M08-/M08.88) | …… other specified site |
| [*M08.89*](https://www.icd10data.com/ICD10CM/Codes/M00-M99/M05-M14/M08-/M08.89) | …… multiple sites |
| [*M08.9*](https://www.icd10data.com/ICD10CM/Codes/M00-M99/M05-M14/M08-/M08.9) | Juvenile arthritis, unspecified |
| [*M08.90*](https://www.icd10data.com/ICD10CM/Codes/M00-M99/M05-M14/M08-/M08.90) | …… unspecified site |
| [*M08.91*](https://www.icd10data.com/ICD10CM/Codes/M00-M99/M05-M14/M08-/M08.91) | Juvenile arthritis, unspecified, shoulder |
| [*M08.911*](https://www.icd10data.com/ICD10CM/Codes/M00-M99/M05-M14/M08-/M08.911) | Juvenile arthritis, unspecified, right shoulder |
| [*M08.912*](https://www.icd10data.com/ICD10CM/Codes/M00-M99/M05-M14/M08-/M08.912) | Juvenile arthritis, unspecified, left shoulder |
| [*M08.919*](https://www.icd10data.com/ICD10CM/Codes/M00-M99/M05-M14/M08-/M08.919) | Juvenile arthritis, unspecified, unspecified shoulder |
| [*M08.92*](https://www.icd10data.com/ICD10CM/Codes/M00-M99/M05-M14/M08-/M08.92) | Juvenile arthritis, unspecified, elbow |
| [*M08.921*](https://www.icd10data.com/ICD10CM/Codes/M00-M99/M05-M14/M08-/M08.921) | Juvenile arthritis, unspecified, right elbow |
| [*M08.922*](https://www.icd10data.com/ICD10CM/Codes/M00-M99/M05-M14/M08-/M08.922) | Juvenile arthritis, unspecified, left elbow |
| [*M08.929*](https://www.icd10data.com/ICD10CM/Codes/M00-M99/M05-M14/M08-/M08.929) | Juvenile arthritis, unspecified, unspecified elbow |
| [*M08.93*](https://www.icd10data.com/ICD10CM/Codes/M00-M99/M05-M14/M08-/M08.93) | Juvenile arthritis, unspecified, wrist |
| [*M08.931*](https://www.icd10data.com/ICD10CM/Codes/M00-M99/M05-M14/M08-/M08.931) | Juvenile arthritis, unspecified, right wrist |
| [*M08.932*](https://www.icd10data.com/ICD10CM/Codes/M00-M99/M05-M14/M08-/M08.932) | Juvenile arthritis, unspecified, left wrist |
| [*M08.939*](https://www.icd10data.com/ICD10CM/Codes/M00-M99/M05-M14/M08-/M08.939) | Juvenile arthritis, unspecified, unspecified wrist |
| [*M08.94*](https://www.icd10data.com/ICD10CM/Codes/M00-M99/M05-M14/M08-/M08.94) | Juvenile arthritis, unspecified, hand |
| [*M08.941*](https://www.icd10data.com/ICD10CM/Codes/M00-M99/M05-M14/M08-/M08.941) | Juvenile arthritis, unspecified, right hand |
| [*M08.942*](https://www.icd10data.com/ICD10CM/Codes/M00-M99/M05-M14/M08-/M08.942) | Juvenile arthritis, unspecified, left hand |
| [*M08.949*](https://www.icd10data.com/ICD10CM/Codes/M00-M99/M05-M14/M08-/M08.949) | Juvenile arthritis, unspecified, unspecified hand |
| [*M08.95*](https://www.icd10data.com/ICD10CM/Codes/M00-M99/M05-M14/M08-/M08.95) | Juvenile arthritis, unspecified, hip |
| [*M08.951*](https://www.icd10data.com/ICD10CM/Codes/M00-M99/M05-M14/M08-/M08.951) | Juvenile arthritis, unspecified, right hip |
| [*M08.952*](https://www.icd10data.com/ICD10CM/Codes/M00-M99/M05-M14/M08-/M08.952) | Juvenile arthritis, unspecified, left hip |
| [*M08.959*](https://www.icd10data.com/ICD10CM/Codes/M00-M99/M05-M14/M08-/M08.959) | Juvenile arthritis, unspecified, unspecified hip |
| [*M08.96*](https://www.icd10data.com/ICD10CM/Codes/M00-M99/M05-M14/M08-/M08.96) | Juvenile arthritis, unspecified, knee |
| [*M08.961*](https://www.icd10data.com/ICD10CM/Codes/M00-M99/M05-M14/M08-/M08.961) | Juvenile arthritis, unspecified, right knee |
| [*M08.962*](https://www.icd10data.com/ICD10CM/Codes/M00-M99/M05-M14/M08-/M08.962) | Juvenile arthritis, unspecified, left knee |
| [*M08.969*](https://www.icd10data.com/ICD10CM/Codes/M00-M99/M05-M14/M08-/M08.969) | Juvenile arthritis, unspecified, unspecified knee |
| [*M08.97*](https://www.icd10data.com/ICD10CM/Codes/M00-M99/M05-M14/M08-/M08.97) | Juvenile arthritis, unspecified, ankle and foot |
| [*M08.971*](https://www.icd10data.com/ICD10CM/Codes/M00-M99/M05-M14/M08-/M08.971) | Juvenile arthritis, unspecified, right ankle and foot |
| [*M08.972*](https://www.icd10data.com/ICD10CM/Codes/M00-M99/M05-M14/M08-/M08.972) | Juvenile arthritis, unspecified, left ankle and foot |
| [*M08.979*](https://www.icd10data.com/ICD10CM/Codes/M00-M99/M05-M14/M08-/M08.979) | Juvenile arthritis, unspecified, unspecified ankle and foot |
| [*M08.98*](https://www.icd10data.com/ICD10CM/Codes/M00-M99/M05-M14/M08-/M08.98) | …… vertebrae |
| [*M08.99*](https://www.icd10data.com/ICD10CM/Codes/M00-M99/M05-M14/M08-/M08.99) | …… multiple sites |
| [*M08.9A*](https://www.icd10data.com/ICD10CM/Codes/M00-M99/M05-M14/M08-/M08.9A) | …… other specified site |

**Supplementary table 2a – ICD-10 codes for Spondyloarthritis (Ankylosing Spondylitis, psoriatic arthritis)**

| [*M45*](https://www.icd10data.com/ICD10CM/Codes/M00-M99/M45-M49/M45-/M45) | **Ankylosing spondylitis** |
| --- | --- |
| [*M45.0*](https://www.icd10data.com/ICD10CM/Codes/M00-M99/M45-M49/M45-/M45.0) | Ankylosing spondylitis of multiple sites in spine |
| [*M45.1*](https://www.icd10data.com/ICD10CM/Codes/M00-M99/M45-M49/M45-/M45.1) | Ankylosing spondylitis of occipito atlanto axial region |
| [*M45.2*](https://www.icd10data.com/ICD10CM/Codes/M00-M99/M45-M49/M45-/M45.2) | Ankylosing spondylitis of cervical region |
| [*M45.3*](https://www.icd10data.com/ICD10CM/Codes/M00-M99/M45-M49/M45-/M45.3) | Ankylosing spondylitis of cervicothoracic region |
| [*M45.4*](https://www.icd10data.com/ICD10CM/Codes/M00-M99/M45-M49/M45-/M45.4) | Ankylosing spondylitis of thoracic region |
| [*M45.5*](https://www.icd10data.com/ICD10CM/Codes/M00-M99/M45-M49/M45-/M45.5) | Ankylosing spondylitis of thoracolumbar region |
| [*M45.6*](https://www.icd10data.com/ICD10CM/Codes/M00-M99/M45-M49/M45-/M45.6) | Ankylosing spondylitis lumbar region |
| [*M45.7*](https://www.icd10data.com/ICD10CM/Codes/M00-M99/M45-M49/M45-/M45.7) | Ankylosing spondylitis of lumbosacral region |
| [*M45.8*](https://www.icd10data.com/ICD10CM/Codes/M00-M99/M45-M49/M45-/M45.8) | Ankylosing spondylitis sacral and sacrococcygeal region |
| [*M45.9*](https://www.icd10data.com/ICD10CM/Codes/M00-M99/M45-M49/M45-/M45.9) | Ankylosing spondylitis of unspecified sites in spine |
| [*M45.A*](https://www.icd10data.com/ICD10CM/Codes/M00-M99/M45-M49/M45-/M45.A) | Non radiographic axial spondyloarthritis |
| [*M45.A0*](https://www.icd10data.com/ICD10CM/Codes/M00-M99/M45-M49/M45-/M45.A0) | …… of unspecified sites in spine |
| [*M45.A1*](https://www.icd10data.com/ICD10CM/Codes/M00-M99/M45-M49/M45-/M45.A1) | …… of occipito atlanto axial region |
| [*M45.A2*](https://www.icd10data.com/ICD10CM/Codes/M00-M99/M45-M49/M45-/M45.A2) | …… of cervical region |
| [*M45.A3*](https://www.icd10data.com/ICD10CM/Codes/M00-M99/M45-M49/M45-/M45.A3) | …… of cervicothoracic region |
| [*M45.A4*](https://www.icd10data.com/ICD10CM/Codes/M00-M99/M45-M49/M45-/M45.A4) | …… of thoracic region |
| [*M45.A5*](https://www.icd10data.com/ICD10CM/Codes/M00-M99/M45-M49/M45-/M45.A5) | …… of thoracolumbar region |
| [*M45.A6*](https://www.icd10data.com/ICD10CM/Codes/M00-M99/M45-M49/M45-/M45.A6) | …… of lumbar region |
| [*M45.A7*](https://www.icd10data.com/ICD10CM/Codes/M00-M99/M45-M49/M45-/M45.A7) | …… of lumbosacral region |
| [*M45.A8*](https://www.icd10data.com/ICD10CM/Codes/M00-M99/M45-M49/M45-/M45.A8) | …… of sacral and sacrococcygeal region |
| [*M45.AB*](https://www.icd10data.com/ICD10CM/Codes/M00-M99/M45-M49/M45-/M45.AB) | …… of multiple sites in spine |
| [*L40.5*](https://www.icd10data.com/ICD10CM/Codes/L00-L99/L40-L45/L40-/L40.5) | **Arthropathic arthritis** |
| [*L40.50*](https://www.icd10data.com/ICD10CM/Codes/L00-L99/L40-L45/L40-/L40.50) | …… unspecified |
| [*L40.51*](https://www.icd10data.com/ICD10CM/Codes/L00-L99/L40-L45/L40-/L40.51) | Distal interphalangeal psoriatic arthropathy |
| [*L40.52*](https://www.icd10data.com/ICD10CM/Codes/L00-L99/L40-L45/L40-/L40.52) | Psoriatic arthritis mutilans |
| [*L40.53*](https://www.icd10data.com/ICD10CM/Codes/L00-L99/L40-L45/L40-/L40.53) | Psoriatic spondylitis |
| [*L40.54*](https://www.icd10data.com/ICD10CM/Codes/L00-L99/L40-L45/L40-/L40.54) | Psoriatic juvenile arthropathy |
| [*L40.59*](https://www.icd10data.com/ICD10CM/Codes/L00-L99/L40-L45/L40-/L40.59) | Other psoriatic arthropathy |
| [*L40.8*](https://www.icd10data.com/ICD10CM/Codes/L00-L99/L40-L45/L40-/L40.8) | Other psoriasis |
| [*L40.9*](https://www.icd10data.com/ICD10CM/Codes/L00-L99/L40-L45/L40-/L40.9) | Psoriasis, unspecified |

**Supplementary table 2b – ICD-10 codes for Systemic Lupus erythematosus**

| [*M32*](https://www.icd10data.com/ICD10CM/Codes/M00-M99/M30-M36/M32-/M32) | **Systemic lupus erythematosus (SLE)** |
| --- | --- |
| [*M32.0*](https://www.icd10data.com/ICD10CM/Codes/M00-M99/M30-M36/M32-/M32.0) | Drug induced systemic lupus erythematosus |
| [*M32.1*](https://www.icd10data.com/ICD10CM/Codes/M00-M99/M30-M36/M32-/M32.1) | Systemic lupus erythematosus with organ or system involvement |
| [*M32.10*](https://www.icd10data.com/ICD10CM/Codes/M00-M99/M30-M36/M32-/M32.10) | Systemic lupus erythematosus, organ or system involvement unspecified |
| [*M32.11*](https://www.icd10data.com/ICD10CM/Codes/M00-M99/M30-M36/M32-/M32.11) | Endocarditis in systemic lupus erythematosus |
| [*M32.12*](https://www.icd10data.com/ICD10CM/Codes/M00-M99/M30-M36/M32-/M32.12) | Pericarditis in systemic lupus erythematosus |
| [*M32.13*](https://www.icd10data.com/ICD10CM/Codes/M00-M99/M30-M36/M32-/M32.13) | Lung involvement in systemic lupus erythematosus |
| *M32.14* | Glomerular disease in systemic lupus erythematosus |
| [*M32.15*](https://www.icd10data.com/ICD10CM/Codes/M00-M99/M30-M36/M32-/M32.15) | Tubulo interstitial nephropathy in systemic lupus erythematosus |
| [*M32.19*](https://www.icd10data.com/ICD10CM/Codes/M00-M99/M30-M36/M32-/M32.19) | Other organ or system involvement in systemic lupus erythematosus |
| [*M32.8*](https://www.icd10data.com/ICD10CM/Codes/M00-M99/M30-M36/M32-/M32.8) | Other forms of systemic lupus erythematosus |
| *M32.9* | Systemic lupus erythematosus, unspecified |

**Supplementary table 2c – ICD-10 codes for  Mixed connective tissue disease**

| *M35.1* | Other overlap syndromes |
| --- | --- |

**Supplementary table 3a – ICD-10 codes for  Systemic Sclerosis**

| [*M34*](https://www.icd10data.com/ICD10CM/Codes/M00-M99/M30-M36/M34-/M34) | Systemic sclerosis [scleroderma] |
| --- | --- |
| [*M34.0*](https://www.icd10data.com/ICD10CM/Codes/M00-M99/M30-M36/M34-/M34.0) | Progressive systemic sclerosis |
| [*M34.1*](https://www.icd10data.com/ICD10CM/Codes/M00-M99/M30-M36/M34-/M34.1) | CR(E)ST syndrome |
| [*M34.2*](https://www.icd10data.com/ICD10CM/Codes/M00-M99/M30-M36/M34-/M34.2) | Systemic sclerosis induced by drug and chemical |
| [*M34.8*](https://www.icd10data.com/ICD10CM/Codes/M00-M99/M30-M36/M34-/M34.8) | Other forms of systemic sclerosis |
| [*M34.81*](https://www.icd10data.com/ICD10CM/Codes/M00-M99/M30-M36/M34-/M34.81) | Systemic sclerosis with lung involvement |
| [*M34.82*](https://www.icd10data.com/ICD10CM/Codes/M00-M99/M30-M36/M34-/M34.82) | Systemic sclerosis with myopathy |
| [*M34.83*](https://www.icd10data.com/ICD10CM/Codes/M00-M99/M30-M36/M34-/M34.83) | Systemic sclerosis with polyneuropathy |
| [*M34.89*](https://www.icd10data.com/ICD10CM/Codes/M00-M99/M30-M36/M34-/M34.89) | Other systemic sclerosis |
| [*M34.9*](https://www.icd10data.com/ICD10CM/Codes/M00-M99/M30-M36/M34-/M34.9) | Systemic sclerosis, unspecified |

**Supplementary table 3b – ICD-10 codes for Inflammatory myopathies**

| [*M33.0*](https://www.icd10data.com/ICD10CM/Codes/M00-M99/M30-M36/M33-/M33.0) | **Juvenile dermatomyositis** |
| --- | --- |
| [*M33.00*](https://www.icd10data.com/ICD10CM/Codes/M00-M99/M30-M36/M33-/M33.00) | …… organ involvement unspecified |
| [*M33.01*](https://www.icd10data.com/ICD10CM/Codes/M00-M99/M30-M36/M33-/M33.01) | …… with respiratory involvement |
| [*M33.02*](https://www.icd10data.com/ICD10CM/Codes/M00-M99/M30-M36/M33-/M33.02) | …… with myopathy |
| [*M33.03*](https://www.icd10data.com/ICD10CM/Codes/M00-M99/M30-M36/M33-/M33.03) | …… without myopathy |
| [*M33.09*](https://www.icd10data.com/ICD10CM/Codes/M00-M99/M30-M36/M33-/M33.09) | …… with other organ involvement |
| [*M33.1*](https://www.icd10data.com/ICD10CM/Codes/M00-M99/M30-M36/M33-/M33.1) | **Other dermatomyositis** |
| [*M33.10*](https://www.icd10data.com/ICD10CM/Codes/M00-M99/M30-M36/M33-/M33.10) | ……organ involvement unspecified |
| [*M33.11*](https://www.icd10data.com/ICD10CM/Codes/M00-M99/M30-M36/M33-/M33.11) | …… with respiratory involvement |
| [*M33.12*](https://www.icd10data.com/ICD10CM/Codes/M00-M99/M30-M36/M33-/M33.12) | …… with myopathy |
| [*M33.13*](https://www.icd10data.com/ICD10CM/Codes/M00-M99/M30-M36/M33-/M33.13) | …… without myopathy |
| [*M33.19*](https://www.icd10data.com/ICD10CM/Codes/M00-M99/M30-M36/M33-/M33.19) | …… with other organ involvement |
| [*M33.2*](https://www.icd10data.com/ICD10CM/Codes/M00-M99/M30-M36/M33-/M33.2) | **Polymyositis** |
| [*M33.20*](https://www.icd10data.com/ICD10CM/Codes/M00-M99/M30-M36/M33-/M33.20) | …… organ involvement unspecified |
| [*M33.21*](https://www.icd10data.com/ICD10CM/Codes/M00-M99/M30-M36/M33-/M33.21) | …… with respiratory involvement |
| [*M33.22*](https://www.icd10data.com/ICD10CM/Codes/M00-M99/M30-M36/M33-/M33.22) | …… with myopathy |
| [*M33.29*](https://www.icd10data.com/ICD10CM/Codes/M00-M99/M30-M36/M33-/M33.29) | …… with other organ involvement |
| [*M33.9*](https://www.icd10data.com/ICD10CM/Codes/M00-M99/M30-M36/M33-/M33.9) | **Dermatopolymyositis, unspecified** |
| [*M33.90*](https://www.icd10data.com/ICD10CM/Codes/M00-M99/M30-M36/M33-/M33.90) | …… organ involvement unspecified |
| [*M33.91*](https://www.icd10data.com/ICD10CM/Codes/M00-M99/M30-M36/M33-/M33.91) | …… with respiratory involvement |
| [*M33.92*](https://www.icd10data.com/ICD10CM/Codes/M00-M99/M30-M36/M33-/M33.92) | …… with myopathy |
| [*M33.93*](https://www.icd10data.com/ICD10CM/Codes/M00-M99/M30-M36/M33-/M33.93) | …… without myopathy |
| [*M33.99*](https://www.icd10data.com/ICD10CM/Codes/M00-M99/M30-M36/M33-/M33.99) | …… with other organ involvement |

**Supplementary table 3c – ICD-10 codes for Sjogren Syndrome**

| [*M35.0*](https://www.icd10data.com/ICD10CM/Codes/M00-M99/M30-M36/M35-/M35.0) | **Sjogren Syndrome** |
| --- | --- |
| [*M35.00*](https://www.icd10data.com/ICD10CM/Codes/M00-M99/M30-M36/M35-/M35.00) | …… unspecified |
| [*M35.01*](https://www.icd10data.com/ICD10CM/Codes/M00-M99/M30-M36/M35-/M35.01) | …… with keratoconjunctivitis |
| [*M35.02*](https://www.icd10data.com/ICD10CM/Codes/M00-M99/M30-M36/M35-/M35.02) | …… with lung involvement |
| [*M35.03*](https://www.icd10data.com/ICD10CM/Codes/M00-M99/M30-M36/M35-/M35.03) | …… with myopathy |
| [*M35.04*](https://www.icd10data.com/ICD10CM/Codes/M00-M99/M30-M36/M35-/M35.04) | …… with tubulo interstitial nephropathy |
| [*M35.05*](https://www.icd10data.com/ICD10CM/Codes/M00-M99/M30-M36/M35-/M35.05) | …… with inflammatory arthritis |
| [*M35.06*](https://www.icd10data.com/ICD10CM/Codes/M00-M99/M30-M36/M35-/M35.06) | …… with peripheral nervous system involvement |
| [*M35.07*](https://www.icd10data.com/ICD10CM/Codes/M00-M99/M30-M36/M35-/M35.07) | …… with central nervous system involvement |
| [*M35.08*](https://www.icd10data.com/ICD10CM/Codes/M00-M99/M30-M36/M35-/M35.08) | …… with gastrointestinal involvement |
| [*M35.0A*](https://www.icd10data.com/ICD10CM/Codes/M00-M99/M30-M36/M35-/M35.0A) | …… with glomerular disease |
| [*M35.0B*](https://www.icd10data.com/ICD10CM/Codes/M00-M99/M30-M36/M35-/M35.0B) | …… with vasculitis |
| [*M35.0C*](https://www.icd10data.com/ICD10CM/Codes/M00-M99/M30-M36/M35-/M35.0C) | …… with dental involvement |
| [*M35.09*](https://www.icd10data.com/ICD10CM/Codes/M00-M99/M30-M36/M35-/M35.09) | …… with other organ involvement |
